# Two-dimensional ferroelectricity in a single-element bismuth monolayer

**DOI:** 10.1038/s41586-023-05848-5

**Published:** 2023-04-05

**Authors:** Jian Gou, Hua Bai, Xuanlin Zhang, Yu Li Huang, Sisheng Duan, A. Ariando, Shengyuan A. Yang, Lan Chen, Yunhao Lu, Andrew Thye Shen Wee

**Affiliations:** 1 Department of Physics, National University of Singapore, Singapore, Singapore; 2 Zhejiang Province Key Laboratory of Quantum Technology and Device, School of Physics, State Key Laboratory of Silicon Materials, School of Materials Science and Engineering, Zhejiang University, Hangzhou, China; 3 Department of Physics, Faculty of Science, Kunming University of Science and Technology, Kunming, China; 4 Joint School of National University of Singapore and Tianjin University, International Campus of Tianjin University, Fuzhou, China; 5 Research Laboratory for Quantum Materials, Singapore University of Technology and Design, Singapore, Singapore; 6 Institute of Physics, Chinese Academy of Sciences, Beijing, China; 7 School of Physics, University of Chinese Academy of Sciences, Beijing, China; 8 Centre for Advanced 2D Materials (CA2DM) and Graphene Research Centre (GRC), National University of Singapore, Singapore, Singapore

**Keywords:** Ferroelectrics and multiferroics, Electronic properties and materials, Two-dimensional materials, Electronic properties and materials, Scanning probe microscopy

## Abstract

Ferroelectric materials are fascinating for their non-volatile switchable electric polarizations induced by the spontaneous inversion-symmetry breaking. However, in all of the conventional ferroelectric compounds, at least two constituent ions are required to support the polarization switching^1,2^. Here, we report the observation of a single-element ferroelectric state in a black phosphorus-like bismuth layer^3^, in which the ordered charge transfer and the regular atom distortion between sublattices happen simultaneously. Instead of a homogenous orbital configuration that ordinarily occurs in elementary substances, we found the Bi atoms in a black phosphorous-like Bi monolayer maintain a weak and anisotropic *sp* orbital hybridization, giving rise to the inversion-symmetry-broken buckled structure accompanied with charge redistribution in the unit cell. As a result, the in-plane electric polarization emerges in the Bi monolayer. Using the in-plane electric field produced by scanning probe microscopy, ferroelectric switching is further visualized experimentally. Owing to the conjugative locking between the charge transfer and atom displacement, we also observe the anomalous electric potential profile at the 180° tail-to-tail domain wall induced by competition between the electronic structure and electric polarization. This emergent single-element ferroelectricity broadens the mechanism of ferroelectrics and may enrich the applications of ferroelectronics in the future.

## Abstract (web summary)

A single-element ferroelectric state is observed in a black phosphorus-like bismuth layer, in which the ordered charge transfer and the regular atom distortion between sublattices happen simultaneously and ferroelectric switching is further visualized experimentally.

## Main

Ferroelectrics are well known for their applications in the non-volatile memories^4^ and electric sensors^5^, and their applications have been extended to the areas of ferroelectric photovoltaics for the efficient renewable energy harvesting^6^ and synaptic devices for the powerful neuromorphic computing^7^. Recently, research on ferroelectrics has been expanded to the two-dimensional (2D) limits with distinct performance^8–10^, including perovskite ferroelectrics at the unit-cell thickness^11,12^, single layer ferroelectrics with in-plane or out-of-plane polarization^13,14^ and 2D moiré ferroelectrics by the van der Waals stacking^15,16^.

Normally, ferroelectric materials are compounds that consist of two or more different constituent elements^1,2^. The electron redistribution during the chemical bond formation instantaneously renormalizes the valence orbitals and yields the anion and cation centres. Further relative distortion, sliding or charge transfer between the positive and negative charge centres in a unit cell produces the ordering of electric dipoles to sustain the ferroelectricity^17–19^. By contrast, as the atoms in a unit cell of an elementary substance are identical, ordered electric dipole or even ferroelectric polarization seem difficult to form spontaneously. The realization of single-element ferroelectricity also lacks experimental demonstration so far. Nevertheless, elements situated between metals and insulators in the periodic table show flexible bonding abilities to adopt several states in one system, such as Sn atoms in the 2D Sn_2_Bi honeycomb structure show binary states^20,21^. Even in elemental boron, ionicity with inter-sublattice charge transfer was found to arise from the different bonding configurations in each sublattice (B_12_ and B_2_)^22^. The subtle balance between metallic and insulating states in these elements is easy to shift by the different sublattice environments so that both states may be realized simultaneously in a unit cell, providing possibilities to produce cations and anions in a unit cell to achieve ferroelectricity in single-element materials. Recently, some theoretical works have been devoted to exploring single-element polarity or ferroelectricity in elemental Si (ref. ^23^), P (ref. ^24,25^), As (ref. ^25^), Sb (ref. ^25,26^), Te (ref. ^27^) and Bi (ref. ^25,26^). In particular, Xiao et al. predicted that the family of group-V single-element materials in a 2D van der Waals form^28^, that is, the monolayer As, Sb and Bi in the anisotropic α-phase structure, have a non-centrosymmetric ground state to support both cations and anions in a unit cell and produce in-plane ferroelectric polarization along the armchair direction.

## Spontaneous symmetry breaking in single BP-Bi layer

**Fig. 1:**
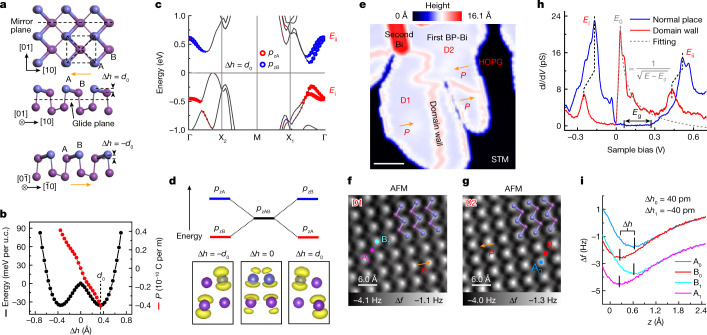
Non-centrosymmetric structure of BP-Bi. **a**, Schematic lattice structure of single layer BP-Bi. Top and side views of Δ*h* = *d*_0_ state are shown in the top and middle panels, respectively. For the Δ*h* = −*d*_0_ state, only a side-view model is shown in the bottom panel. The topmost Bi atoms are coloured light blue to only guide the eye for a better comparison with AFM images. **b**, Calculated free energy per unit cell (u.c.) and polarization *P* versus the buckling Δ*h* show an anharmonic double-well potential and nearly linear relation, respectively. **c**, Band structure of BP-Bi when Δ*h* adopts the right minimum (*d*_0_) of the double-well potential in **b**. The size of the red (blue) circles represents the contributions of the *p*_z_ orbital of sublattice A(B). **d**, Illustration of the revolution of *p*_z_ orbitals at sublattice A and B (top panel). Projected *p*_z_ valence charge density corresponding to three buckling conditions (Δ*h* = −*d*_0_, Δ*h* = 0, Δ*h* = *d*_0_) are shown in the bottom panel. **e**, STM image of BP-Bi on HOPG (*V* = 0.2 V, *I* = 10 pA). Scale bar, 10 nm. **f**,**g**, AFM images of two domains (D1 (**f**) and D2 (**g**)). Ball-and-stick models of the top two layers are superimposed to highlight the atom position. **h**, d*I*/d*V* measured at the domain area and a domain wall (head-to-head, *V* = 0.7 V, *I* = 1.2 nA). The density-of-states (DOS) maximum corresponding to the *p*_z_ bands at Γ point is labelled as *E*_i_ and *E*_ii_. **i**, Δ*f*(*z*) spectra measured on the two sublatticed in **f** and **g**. A_0_ and B_0_ are up-shifted by 2 Hz for clarity. Vertical short bars mark the turn points of the Δ*f*(*z*) curves. Tip height *z* = −260 pm (**f**,**g**,**i**) relative to the height determined by the setpoint *V* = 100 mV, *I* = 10 pA above the BP-Bi surface.

**Extended Data Fig. 1:**
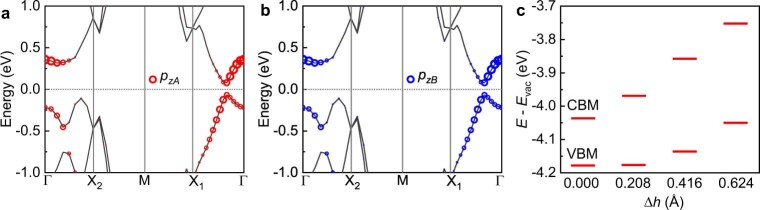
Buckling dependent electronic properties of BP-Bi. **a**,**b**, Band structure of BP-Bi with no atomic buckling (Δ*h* = 0). The size of the red (blue) circles represents the contributions of the *p*_z_ orbital of sublattice A (B). The identical distribution of *p*_zA_ and *p*_zB_ orbital illustrates the degeneracy of A and B sublattice due to the centrosymmetric atomic structure. **c**, Band-edge positions of infinite periodic BP-Bi with different atomic buckling (Δ*h*) at free-standing mode.

The monolayer α-phase Bi has a lattice structure similar to black phosphorous^3,29^, and will be referred to black phosphorous-like-Bi (BP-Bi) thereafter. Owing to the ultra-large atomic number, Bi has a weak hybridization between the 6*s* and 6*p* orbitals so that it features partial *sp*^2^ character other than the homogenous tetrahedral *sp*^3^ configuration that exists in the black phosphorus^26,28^. This brings in a small buckling (Δ*h*) between the neighbouring sublattices with the loss of centrosymmetry^30,31^. As shown in Fig. 1a, the breaking of inversion symmetry allows BP-Bi to adopt two domain states, either the Δ*h* = *d*_0_ or Δ*h* = −*d*_0_ state. Our first-principles calculations reveal the two states can be switched to each other by crossing a small energy barrier of 43 meV per unit cell (Fig. 1b). Moreover, the buckling degree of freedom enlarges the band gap and lifts the degeneracy of the *p*_z_ orbitals at sublattice A and B (Extended Data Fig. 1). Taking Δ*h* = *d*_0_ for instance (Fig. 1c, Δ*h* adopts the right minimum of the double-well potential), the valence band and conduction band at the Γ point are mainly contributed by the *p*_z_ orbitals of A and B sublattice, respectively. When the Fermi level crosses the band gap, the valence *p*_z_ orbitals at the A sublattice are fully occupied, and the *p*_z_ orbitals at the B lattice are empty. In real space, this corresponds to electron transfer from sublattice B to sublattice A, leading to a spontaneously polarized character (Fig. 1d). The anharmonic double-well potential with a small barrier implies the possible ferroelectric switch between the two domain states. The nearly linear dependence between the polarization (*P*) and the buckling degree (Δ*h*), along with the mirror (glide) symmetry refer to the (01) surface (in-plane central surface) (Fig. 1a), indicate that Δ*h* is an order parameter to characterize the ferroelectricity of BP-Bi.

**Extended Data Fig. 2:**
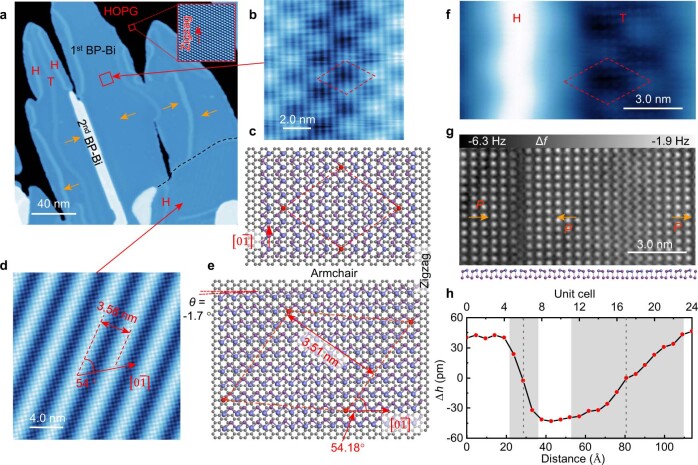
Moiré pattern and domain wall in BP-Bi single layer. **a**, STM image of BP-Bi island that contains two different ferroelectric domain walls, partly marked by H (head-to-head domain wall) and T (tail-to-tail domain wall) (setpoint: *V* = 0.4 V, *I* = 10 pA). **b**,**d**, Zoom-in STM images of two typical moiré patterns formed by aligning the $[0\bar{1}]$ of BP-Bi to the zigzag direction (**b**) or armchair direction (**d**) of the graphite substrate with a small angle *θ* (setpoint: **b**, *V* = 0.1 V, *I* = 100 pA; **d**, *V* = −0.1 V, *I* = 100 pA). Their stacking models are shown in (**c**) and (**e**), respectively. **f**,**g**, STM (**f**) and AFM (**g**) images show the closest 180° head-to-head (H) and tail-to-tail (T) domain walls in the same area (setpoint: **f**, *V* = 0.3 V, *I* = 10 pA). **h**, Buckling (Δ*h*) measurement perpendicularly crossing H and T domain wall in (**g**). Tip height *z* = −230 pm in (**g**,**h**) relative to the height determined by the setpoint *V* = 100 mV, *I* = 10 pA above normal Bi surface. All the orange arrows in (**a**) and (**g**) denote the direction of polarization.

**Extended Data Fig. 3:**
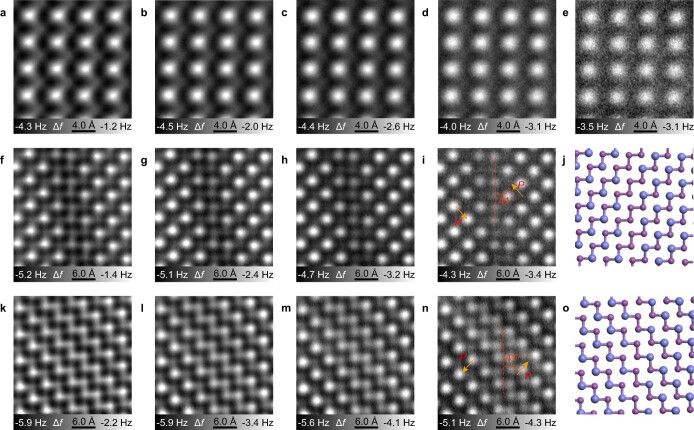
Tip height dependent AFM imaging. **a**–**e**, AFM images of a normal area measured at tip height *z* = −290 pm (**a**), −270 pm (**b**), −250 pm (**c**), −230 pm (**d**), −200 pm (**e**). The atomic model to this structure can be found in Fig. 1a in the main text. **f**–**i**, AFM images of a 41° inclined 180° head-to-head domain wall measured at tip height *z* = −270 pm (**f**), −250 pm (**g**), −230 pm (**h**), −210 pm (**i**). **j**, Schematic ball-and-stick model illustrates the top two layers of the atomic structure in (**f**–**i**). **k**-**n**, Atomic AFM images of the 41° inclined 180° tail-to-tail domain wall measured at tip height *z* = −270 pm (**k**), −250 pm (**l**), −230 pm (**m**), −210 pm (**n**). **o**, Schematic ball-and-stick model demonstrates the top two layers of atomic structure in (**k**–**n**). The topmost atoms are coloured to light blue to guide the eye. *z* = 0 pm is defined by the setpoint of *V* = 100 mV, *I* = 10 pA above normal Bi surface. All the schematics in (**j**) and (**o**) do not reflect the real relative height, as the buckling is gradually changing near the wall.

Experimentally, we grew BP-Bi on highly oriented pyrolytic graphite (HOPG). Figure 1e shows a scanning tunnelling microscopy (STM) image of the monolayer BP-Bi with some second layer nanoribbons along the $[0\bar{1}]$ direction (Extended Data Fig. 2a)^32^. The atomic-resolved non-contact atomic force microscopy (AFM) measurement indicates two different states in two neighbouring domains separated by a domain wall (Fig. 1f,g and Extended Data Figs. 3f–o and 8). We performed force spectroscopy measurements (the Δ*f*(*z*) spectra) to find out the relative distance between the Bi atoms and tip apex^33^. At the constant-height mode, the measured height difference of the turning points of the Δ*f*(*z*) spectra on sublattice A and B in Fig. 1i quantitatively present the buckling degree, and the result Δ*h*_0_ = −Δ*h*_1_ *=* 40 pm is determined (Extended Data Fig. 5a–d). The d*I*/d*V* spectrum at the domain wall shows the *p*_z_ bands (*E*_i_ and *E*_ii_) moving to a higher binding energy compared to the normal domain position, and a sharp peak occurs in the band gap (Fig. 1h), which will be elaborated in detail later.

## In-plane polarization

**Fig. 2:**
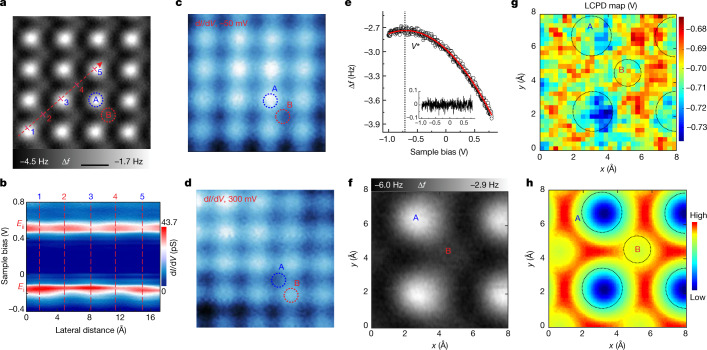
Electron redistribution at A and B sublattice of monolayer BP-Bi. **a**, AFM image of a monolayer BP-Bi. Red and blue dotted circles highlight two atoms in the two sublattices. Scale bar, 4.0 Å. **b**, Constant-height d*I*/d*V* spectra obtained along the red dashed arrow in **a**. **c**,**d**, Constant-height d*I*/d*V* mapping of the occupied states (**c**) and empty states (**d**) in the same area as **a**. **e**, Typical frequency shift (Δ*f*) curve as a function of sample bias is measured above the Bi atoms at the constant-height mode (black circles). Red parabolic fitting (red solid line) yields the V* at the maximum to represent the LCPD. The inset shows the uniform fitted residual. **f**, AFM image of the normal BP-Bi contains four topmost A atoms and one B atom in the middle. **g**, LCPD grid map (30 × 30) measured at the area of **f** illustrates the localized potential difference between the A and B sublattices. **h**, Simulated electrostatic potential above the same structure at a distance of 3 Å from the top Bi plane. Dotted circles in **g** and **h** mark the position of A and B atoms. Tip heights *z* = −260 pm (**a**), −100 pm (**b**), −150 pm (**c**,**d**), −230 pm (**f**) and −100 pm (**e**,**g**), relative to the height determined by the setpoint *V* = 100 mV, *I* = 10 pA above the BP-Bi surface.

In the buckling structure, the charge transfer between the sublattice A and B of BP-Bi is represented by the energy splitting of the *p*_z_ orbitals and the variation of the surface electrostatic potential between A and B. According to the calculated band structure in Fig. 1c, the d*I*/d*V* peak corresponding to *p*_z_ orbitals should be below zero (occupied state, *E*_i_) for one sublattice but above zero (empty state, *E*_ii_) for the other sublattice. Figure 2b shows the d*I*/d*V* cascade along the red dashed arrow (across an ABABA lattice) in Fig. 2a, revealing two traces of peaks. The valence band peak *E*_i_ is clearly strong at the A sublattice (that is, points 1, 3, 5) but weak at the B sublattice (points 2, 4), whereas the conduction band peak *E*_ii_ shows the opposite behaviour. At the 2D scale, the d*I*/d*V* mapping of the valence band (Fig. 2c) and conduction band (Fig. 2d) show the same feature: filled *p*_z_ orbitals localize at the A sublattice, while the empty *p*_z_ orbitals localize at the B sublattice, confirming the predicted electron transfer from B to A.

Another piece of evidence to prove electron transfer is the disparity in the local contact potential difference (LCPD) on each sublattice at atomic scale^34,35^. Figure 2e presents a typical Kelvin probe force microscopy (KPFM) measurement implemented by recording the frequency shift as a function of the sample bias voltage. The electrostatic force caused by the contact potential difference (CPD) between the sample and tip changes by sweeping the bias voltage. When the CPD is totally compensated by the bias (*V*_CPD_ = V^*^), the electrostatic force reaches the minimum, corresponding to the parabolic apex in the frequency shift curve in Fig. 2e. By recording the bias voltage V^*^ site by site in a lateral grid, the surface potential is mapped out and shown in Fig. 2g. AFM was performed simultaneously to determine the atomic structure at the same area (Fig. 2f). It is obvious that the two sublattices A and B have different surface potentials. Using the spontaneous buckled model (Fig. 1a), we calculated the electrostatic potential shown in Fig. 2h, which reproduces the experimental LCPD map and indicates the electron enrichment at the topmost A sublattice. Ultimately, on the basis of the above d*I*/d*V* and LCPD measurement combined with the in-plane distorted atom structure, the in-plane polarization can be confirmed.

## Ferroelectric switching

**Fig. 3:**
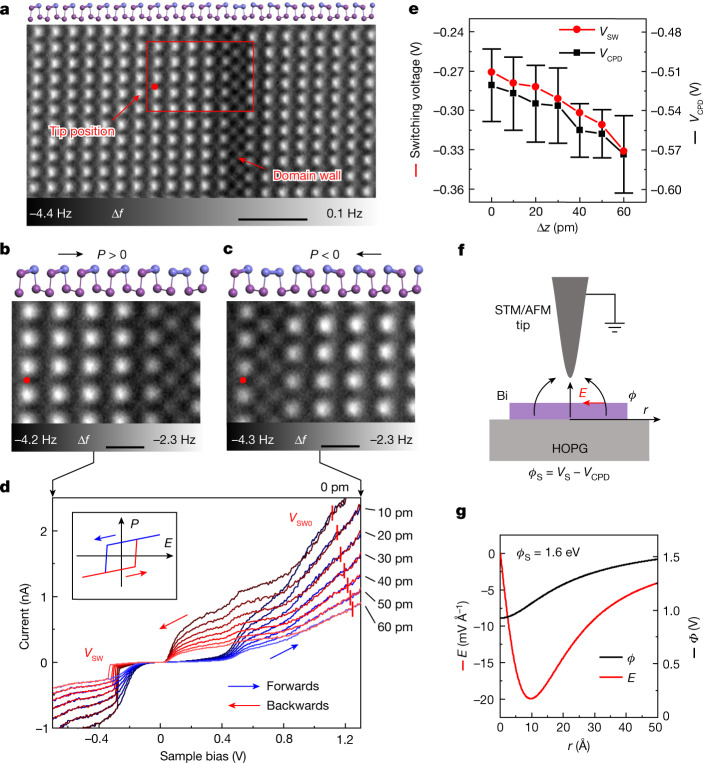
Ferroelectric domain switching by STM/AFM tip. **a**, AFM image of a 180° head-to-head domain wall of monolayer BP-Bi. Scale bar, 20 Å. **b**,**c**, AFM images of the same area marked by the red rectangle in **a**, show the reversal switching before (**b**) and after (**c**) the manipulation. Scale bars, 6.0 Å. The red dots mark the location of tip during switching. The side-view models are put in the upper panels of (**a**–**c**). **d**, Series of *IV* curves with the sample bias sweeping at different tip-sample distances (from Δ*z* = 0 to 60 pm). The inset schematically shows how the polarization of BP-Bi changes with electric field. Red vertical bars mark the switching voltage at the positive bias side. **e**, Tip-height dependent switching voltage (*V*_SW_) and measured CPD (*V*_CPD_). Error bars represent the standard deviation from several measurements. **f**, Schematic diagram of the electric field produced by the STM/AFM tip. **g**, Calculated electric potential and in-plane electric field on the BP-Bi surface. Tip height *z* = −260 pm (**a**), −250 pm (**b**,**c**) and the initial tip height (Δ*z* = 0 pm) in **d**,**e** is −50 pm relative to the height determined by the setpoint *V* = 100 mV, *I* = 10 pA above the BP-Bi surface.

The polarization of a ferroelectric material can be reversed by an applied electric field^36^. In our experiments, we use the in-plane component of electric field from the conductive STM/AFM tip to switch the polarization of small ferroelectric domains close to the tip^37^. To facilitate the switching, we set the tip near a domain wall so that the size of the switched domain is comparable to the limited range of the electric field produced by the tip end (Fig. 3a). During the sample bias sweeping at a specific tip height, the *IV* spectra are recorded as shown in Fig. 3d. When the voltage ramps from negative to positive bias, a low conductance appears at around 0.2 V due to the band gap of BP-Bi under the tip (blue series curves). Continuous bias increase causes a small current jump labelled by the red vertical bars. While sweeping the voltage back (from positive to negative), a larger hysteretic current is maintained with a substantial conductance emerging at around 0.2 V in the gap (red series curves). According to the d*I*/d*V* curves of the domain wall (Fig. 1h), the large in-gap current indicates the domain wall has been moved to the tip position during the application of the positive bias. When the bias voltage reaches a negative value *V*_SW_, the current jumps to the original level, suggesting the domain wall is moved back. The AFM images (Fig. 3b,c) after the forwards and backwards voltage sweeping demonstrate the movement of the domain wall directly. Figure 3c shows the domain wall movement to the left side with a distance of four lattice periods after the forwards manipulation, and Fig. 3b shows the domain wall moving back to the original position after changing the bias backwards to below *V*_SW_. Accordingly, the ferroelectric switching between the two domain states (Δ*h* = *d*_0_ and Δ*h* = −*d*_0_) is experimentally verified. The domain wall in Fig. 3a can be assigned to the 180° head-to-head ferroelectric domain wall with the polarization labelled in Fig. 3b,c.

In the hysteretic domain manipulation, the switching voltage at both positive bias side (*V*_SW0_) and negative bias side (*V*_SW_) show tip-height dependence. When the relative tip height Δ*z* rises from 0 to 60 pm, the switching voltage *V*_SW0_ at the positive bias side is found to increase while *V*_SW_ at the negative bias decreases (Fig. 3d,e). We also measured the LCPD at different heights (*V*_CPD_) (Fig. 3e), which reveals a gradual LCPD decrease with the tip height (Δ*z*) climb due to the semimetallicity of graphite^38^. The tip in our setup is grounded with a bias voltage (*V*_S_) applied to the sample, the electric potential of the HOPG can be written as *Φ*_S_ = *V*_S_ − *V*_CPD_. Thus, the increase of tip height strengthens the electric field under the tip through the decrease of the *V*_CPD_, but weakens the electric field by the rise of the tip-sample distance. Nonetheless, because *Φ*_S_ is small at the negative bias side and large at the positive bias side, we find the electric field is dominated by the LCPD at the negative bias side (*V*_SW_), and dominated by the tip-sample distance at the positive bias side (Extended Data Fig. 7g,h). Therefore, the switching bias required to trigger the same domain switch at a higher tip height has to decrease at the negative bias side but increasing at the positive bias side accordingly, which also explains the correlated variation between *V*_SW_ and *V*_CPD_ in Fig. 3e.

Quantitatively, the electric potential and electric field below the tip are derived by solving the Poisson equation in prolate spheroidal coordinates. The switch bias at the positive bias side was set to *V*_S_ = 1.0 V (approximately *Φ*_S_ = 1.6 eV). The calculated results are shown in Fig. 3g. Differentiating the electric potential on the BP-Bi surface (*Φ*) produces an in-plane electric field of as large as −20 mV Å^−1^ at the distance of roughly 10 Å to the centre (*r* = 0 Å). On the other hand, we can estimate the coercive field by ${E}_{{\rm{c}}}=2\sqrt{-{\alpha }^{3}/27\beta }$, where *α* = −8.93 × 10^−18^ F^−1^ and *β* = 4.25 × 10^−39^ m^2^ C^−2^ F^−1^ are the coefficients of the second- and fourth-order terms in the Landau model fitted to the density functional theory- (DFT-) derived double-well potential in Fig. 1b (ref. ^36^). The estimated *E*_c_ = 15.7 mV Å^−1^ is comparable to the calculated in-plane electric field above (Fig. 3g). This may be understood that the highly localized in-plane electric field works mainly on the domain part between tip and wall, thus demanding that the electric field overcomes *E*_c_. It is noticeable that they are also close to that in the reported ferroelectric polymer PVDF^39^ and strained transition metal oxides^40^^,^^41^. As spontaneous polarization (*P*_s_) is determined by the same *α* and *β* through ${P}_{{\rm{s}}}=\sqrt{-\alpha /\beta }$, the consistency between *E*_c_ and in-plane electric field *E* in our experiment also verifies the DFT result *P*_s_ = 0.41 × 10^−10^ C per m (Fig. 1b), which resembles the polarization of monolayer SnTe (roughly 1 × 10^−10^ C per m)^42^.

## 180° domain walls

**Fig. 4:**
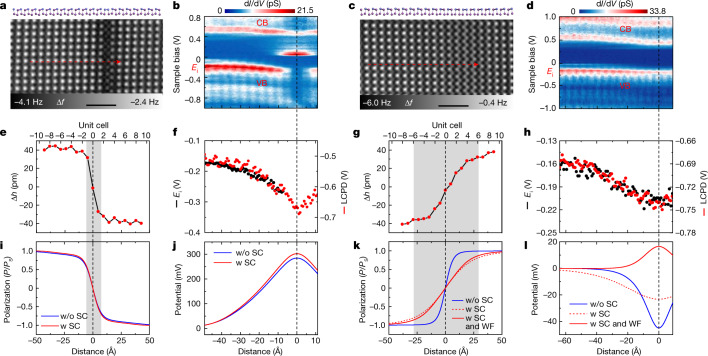
Domain width and band bending at the 180° domain walls. **a**,**c**, AFM images of the 180° head-to-head domain wall (**a**) and tail-to-tail domain wall (**c**) with the side-view models in respective upper panels. Scale bars, 20 Å. **b**,**d**, d*I*/d*V* line maps perpendicularly cross the domain walls along the red dashed arrows in **a** and **c** show the band evolution of the head-to-head (**b**) and tail-to-tail (**d**) domain wall. **e**,**g**, Experimental buckling degree of Bi atoms around the head-to-head domain wall (**e**) and tail-to-tail domain wall (**g**). **f**,**h**, Band bending of the head-to-head domain wall (**f**) and tail-to-tail domain wall (**h**) measured by tracing the *E*_i_ peak in the d*I*/d*V* line maps (black dots), and LCPD measurement (red dots). **i**–**l**, The calculated order parameter (**i**,**k**) and electric profile (**j**,**l**) of the head-to-head (**i**,**j**) and tail-to-tail (**k**,**l**) domain wall with (w, red) or without (w/o, blue) considering the screened Coulomb interaction (SC) on the basis of the thermodynamic theory. The red solid curves in (**k**,**l**) are the calculated results containing the buckling-dependent work function variation (WF) in the tail-to-tail domain wall. The shadow areas in **e**, **g**, **i** and **k** highlight the width of respective domain walls. Wall width is defined by |Δ*h*| < *d*_0_ × tanh(1). Tip heights *z* = −250 pm (**a**), −50 pm (**b**,**f**), −260 pm (**c**), −60 pm (**d**,**h**) and −290 pm (**e**,**g**), relative to the height determined by the setpoint *V* = 100 mV, *I* = 10 pA above the normal BP-Bi surface.

Besides the 180° head-to-head domain wall in the monolayer BP-Bi (Figs. 1e, 3a, 4a and Extended Data Fig. 9), we also observe the conjugated 180° tail-to-tail domain wall (Fig. 4c). The AFM measurements show that the 180° head-to-head domain wall has a wall width of about 15 Å (three unit cells, shadow area in Fig. 4e), whereas the 180° tail-to-tail domain wall has a wider width of roughly 56 Å (12 unit cells, shadow area in Fig. 4g). The d*I*/d*V* measurements of the head-to-head domain wall reveal a one-dimensional electronic state in the band gap, and both conduction band and valence band near the wall bend to a higher binding energy (Fig. 4b), indicating the accumulation of electrons around the wall. A close determination of the band bending by extracting *E*_i_ peak and measuring the LCPD by KPFM show the same bending profile and a total band bending of about 170 meV (Fig. 4f). When turning to the tail-to-tail domain wall, the d*I*/d*V* spectra, however, show the incongruous band movement in the valence band and conduction band (Fig. 4d). The same band bending assessment methods by the *E*_i_ analysis and LCPD measurement in Fig. 4h suggest a smaller but identical bending direction to the head-to-head domain wall (Fig. 4f).

**Extended Data Fig. 4:**
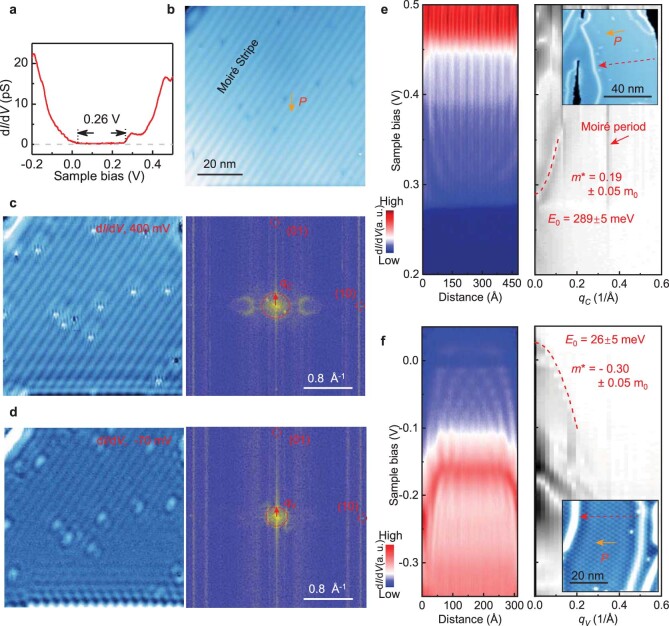
Band structure measurement of BP-Bi near the Fermi surface. **a**, A typical d*I*/d*V* spectrum acquired on the BP-Bi shows a band gap above the Fermi surface (initial setpoint: *V* = 500 mV, *I* = 1.0 nA). **b**, STM image (*V* = −70 mV, *I* = 150 pA) of a single domain area. **c**,**d**, d*I*/d*V* maps (left panel) and Fourier-transformed d*I*/d*V* maps (right panel) of conduction band (**c**) and valence band (**d**) are measured at the area in (**b**). The atomic Bragg peaks are highlighted by the red dashed circles. **e**,**f**, d*I*/d*V* line maps (left panel) and corresponding energy-resolved Fourier transformation (right panel) for the conduction band (**e**) and valence band (**f**) along the armchair direction (initial setpoint: *V* = 500 mV, *I* = 1.3 nA (**e**); *V* = −350 mV, *I* = 1.0 nA (**f**)). The parabolic fittings and trajectories (red dashed lines) for the d*I*/d*V* measurements at VBM and CBM are shown in corresponding insets (*V* = 400 mV, *I* = 15 pA (**e**); *V* = 450 mV, *I* = 15 pA (**f**)).

To understand the experimental observation above, we calculate the order parameter profile at the domain wall using the Landau–Ginzburg–Devonshire theory^43,44^. Considering the intrinsic p-type doping (Extended Data Fig. 4), the screened Coulomb interaction is included to take into account the screening effect of charge carriers on the ferroelectric instability^45^. Figure 4i–l shows the calculated wall width and surface potential profile in the 180° head-to-head and tail-to-tail domain walls. It is apparent that there is nearly no difference between the cases with or without the screened Coulomb interaction in the head-to-head domain wall (Fig. 4i,j). The wall width is about three unit cells and the surface potential increase exponentially at the domain wall, consistent with the experimental observations (Fig. 4e,f). However, for the tail-to-tail domain wall, the wall width and the surface potential have totally different behaviours regarding the cases with and without a screened Coulomb interaction (Fig. 4k,l). For the case without a screened Coulomb interaction, the tail-to-tail domain wall has a similar narrow width (three unit cells) as the head-to-head domain wall. Involving the screened Coulomb interaction results in a wall width four times larger (12 unit cells), which matches the experimental observations.

As demonstrated, BP-Bi is heavily p-type doped (Extended Data Fig. 4). The upwards band bending at the tail-to-tail domain wall in principle will increase the local carrier concentration drastically, which accordingly decrease the Thomas–Fermi screening length and strongly screen the Coulomb interaction or depress the spontaneous polarization *P*_s_ (refs. ^46,47^). Generally, the wall width can be simplified as ${D}_{{\rm{W}}}=4\sqrt{k/2{P}_{{\rm{s}}}^{2}\beta }$ (*k* is the gradient coefficient)^36^, thus the rise of charge carrier density at the domain wall broadens the wall width and flattens the surface potential at tail-to-tail domain wall (red dashed lines in Fig. 4k,l). By contrast, the band bending at the head-to-head domain wall makes the Fermi level shift to the middle of the band gap and lessens the carrier density, which consequently narrows the wall width (Fig. 4i). However, as the screening length at low carrier density is notably larger than the efficient range of the Coulomb interaction^45^, the screening effect changes little with the continued reduction of the carrier concentration, resulting in a slight width shrinking at the head-to-head domain wall.

In addition, the evolution of the electronic structure correlated to the atomic buckling (Δ*h*) at the tail-to-tail domain wall is discussed. Early theoretical and experimental research showed that the band structure is highly buckling dependent^30,48^. Our refined DFT calculations suggest that the position of the Fermi level decreases monotonously with the buckling reduction (Extended Data Fig. 1c), which implies an increase in the work function or gradual charge transfer between the substrate and BP-Bi layer at the wide tail-to-tail domain wall. With this consideration, the amended calculation of the surface potential reverses at the tail-to-tail domain wall, and acts as a similar band bending as that of the head-to-head domain wall (red solid curve in Fig. 4l). This result reproduces the electric potential measurements (Fig. 4h) and illustrates the conjugative correlations between electronic structure and ferroelectric distortion in this monolayer BP-Bi system.

## Conclusion

In this work, we experimentally confirmed in-plane polarization and observed ferroelectric switching in a single-element BP-Bi monolayer, demonstrating the capability to realize ferroelectric polarization in an elementary substance or single-element compound^22^ (for example, bismuth bismuthide here). The spontaneous charge redistribution and the ferroelectric atomic distortion in the BP-Bi show the intercorrelation between the electronic structure and inversion symmetry. Owing to the heavily p-type doping of BP-Bi, the screened Coulomb interaction from the carrier density and the electronic modulation by the ferroelectric distortion were observed at the 180° tail-to-tail domain wall. The single-element ferroelectricity inspires the advantages of ferroelectrics to electrically modulate the band structure, as well as other potential emergent phenomena such as topology and superconductivity beyond magnetism^19^.

## Methods

### Sample preparation and characterization

Single layer BP-Bi was prepared in an ultra-high vacuum molecular-beam epitaxy chamber that connected to the Omicron LT-STM system (1 × 10^−10^ mbar). HOPG was first cleaved in air and then immediately loaded into the molecular-beam epitaxy chamber to degas the surface contamination. Bismuth with a purity of 99.999% was evaporated by a K-cell to the substrate that kept at room temperature. All the STM or scanning tunnelling spectroscopy (STS) and AFM/KPFM measurements were performed at 4.3 K with a tungsten tip glued to the qPlus AFM sensor, except the variable temperature experiment reached the respective temperature by the liquid nitrogen cryostat cooling and resistance counter heating. The clean and atomically sharp tip was obtained by repeat voltage pulses and controllable indentations on the Au(111) substrate. Further vertical CO molecule manipulation was carried out on the same surface to get a CO functionalized apex^49^. Differential conductance (d*I*/d*V*) was obtained by a lock-in technique with a 3–10-mV and 963-Hz modulation superimposed on the sample bias. The AFM sensor with a resonance frequency of 27 kHz and a quality factor of 30,000–70,000 was excited to an amplitude of 40 pm for the AFM measurement and 60 pm for the KPFM measurement. All the images have been processed and rendered using MATLAB and WSxM software^50^.

### First-principles calculations

First-principle calculations were carried out within the DFT formalism, as implemented in the Vienna Ab initio Simulation Package^51^. The local density approximation was used for exchange-correlation functional with a projector augmented-wave^52^ pseudopotentials, treating Bi 5*d* 6*s* 6*p* as valence electrons. The cut-off of the plane wave basis was set above 500 eV to guarantee that the absolute energies are converged to 1 meV. Grimme’s method^53^ was used to incorporate the effects of van der Waals interactions in all geometry optimizations. The vacuum region was set to larger than 25 Å to minimize artificial interactions between images. The 2D Brillouin zone was sampled by a Monkhorst–Pack^54^ *k*-point mesh and it is 12 × 12 for the unit cell. The positions of atoms were fully relaxed until the Hellmann–Feynman force on each atom was less than 0.01 eV Å^−1^. The convergence criteria for energy were set to 10^−5^ eV. The electronic-structure calculations were performed within the scalar relativistic approximation, including a spin-orbit coupling effect. Hybrid functional (HSE06)^54^ was also used to confirm the band gap. All energy levels were calibrated by the corresponding core levels.

### Moiré pattern and domain wall

Owing to the twofold symmetry of the BP-Bi, the moiré patterns produced by stacking single layer BP-Bi on HOPG can be classified into two categories: in type 1, the zigzag BP-Bi ($[0\bar{1}]$ direction) aligns at a small twist angle *θ* with respect to the zigzag direction of graphite; in type 2, the zigzag BP-Bi aligns at a small twist angle *θ* with respect to the armchair direction of graphite. Among them, the ±1.7° type 2 (*θ* = ±1.7°) moiré pattern is unique for the homogeneous stacking sequence between BP-Bi and graphite along the short axis of the moiré superlattice (Extended Data Fig. 2e), which therefore shows the stripe contrast in the STM measurement (Extended Data Fig. 2d). In this kind of moiré pattern, the domain wall is constrained to along the stripe direction owing to the corresponding stripe-like strain and potential distribution in the moiré pattern (Extended Data Fig. 8a and the bottom-right part of Extended Data Fig. 2a). Conversely, the type 1 moiré pattern has the gradually changing stacking sequence along both periodic directions (Extended Data Fig. 2b,c), thus allows the 180° domain wall with an incline angle to exist (Extended Data Fig. 2a,f,g).

For all the domain walls, there are two critical angles that are used to describe them adequately: the angle between polarization vectors of two neighbouring domains and the smallest angle between the domain wall and the polarization vector nearby. We name the last one as the incline angle and only use this part ahead of the domain’s name when the front one is 180°. But we neglect the prefix when the incline angle is 90°, because this kind of domain wall (90° inclined 180° head-to-head or tail-to-tail domain wall) is the most frequently found (Figs. 3 and 4 and Extended Data Figs. 2a and 9). Extended Data Fig. 8 shows some uncommon domain walls in the measurement, they are charged 54° inclined 180° head-to-head domain wall (Extended Data Fig. 8a), neutral 90° head-to-tail domain wall (Extended Data Fig. 8b) and charged 90° head-to-head domain wall (Extended Data Fig. 8c).

### Band edge and band gap

From the d*I*/d*V* measurement on BP-Bi domain area, it is easy to identify a band gap with the valence band maximum (VBM) near the Fermi level and the conduction band minimum (CBM) at around 0.27 eV. To determine the band structure and band edge precisely, we measured the quasiparticle interference (QPI) of the VBM and CBM at the spatial domain and energy domain (Extended Data Fig. 4). The Fourier transform of the 2D STS maps at both valence band and conduction band show the intravalley scattering characterized by the *q*_*c*_ and *q*_v_ in the centre of the reciprocal space. Fourier transform–STS along the high symmetry direction (Γ-X_2_) show the parabolic dispersion at both valence band and valence band near the Fermi surface. The parabolic fittings of the QPI yields the VBM and CBM to be at 26 ± 5 and 289 ± 5 meV, respectively, which confirms a band gap of roughly 260 meV and the intrinsic hole doping in the BP-Bi.

### Buckling determination and edge reconstruction

**Extended Data Fig. 5:**
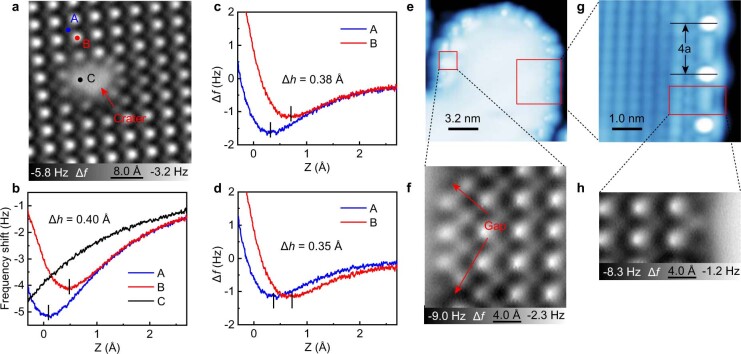
Buckling and edge measurement of BP-Bi. **a**, AFM image of a sub-nanometer scale crater in the BP-Bi. **b**, Δ*f*(*z*) spectra measured at the locations of A, B and C in (**a**). **c**, The Δ*f*(*z*) spectra of A and B after subtracting the background curve C. **d**, Δ*f*(*z*) spectra of A and B after further background force calibration. **e**, STM image of monolayer BP-Bi that contains both types of zigzag edges (setpoint: *V* = −0.2 V, *I* = 10 pA). **f**–**h**, Atomic-resolved AFM (**f**,**h**) and STM (**g**) images show the reconstructed atom arrangement at both sides (setpoint: **g**, *V* = 0.2 V, *I* = 100 pA). Tip height *z* = −240 pm (**a**), −250 pm (**b**), −280 pm (**f**,**h**), relative to the height determined by the setpoint *V* = 100 mV, *I* = 10 pA above normal Bi surface.

Extended Data Fig. 5 shows the AFM measurement of a crater and two different zigzag edges in the BP-Bi. Although the crater is so small that the edges are very short, the reconstruction can be easily confirmed on the atomic scale. Further inspection of the long straight zigzag edges in a BP-Bi ribbon island also shows the same results. The reconstructed gaps and 4a superperiod are responsible for the missing of regular band bending at both zigzag edges (Supplementary Information)^55^.

The buckling (Δ*h*) of the BP-Bi can be roughly determined by obtaining the height difference of the turning points of the Δ*f*(*z*) spectra that were measured above the A and B sublattice. A more precise measurement requires the subtraction of the background force. Here we use the force spectroscopy that was obtained at the crater as the van der Waals force background (curve C). Removing this term gives a smaller buckling value (Δ*h* = 0.38 Å) but still a different frequency shift at the turning point of the two Δ*f*(*z*) spectra, which means the background force at A and B is different. This can be understood to provide the information that the nearest and next-nearest neighbours of A and B have different *z* coordinates and, the A and B atoms have different electrostatic charges. At the tip–sample distance in our experiment, assuming that the electrostatic charge difference is neglectable and the background force for A and B has the same form *F*_b_(*z*), thereof the (subtracted) net force for atom A and B can be written as *F*_A0_(*z*) = *F*_A_(*z*) − *F*_b_(*z* − Δ*h*) and *F*_B0_(*z*) = *F*_B_(*z*) − *F*_b_(*z*), respectively. Because A and B are the same Bi element, curves *F*_A0_(*z*) and *F*_B0_(*z*) should be identical by a translation of Δ*h* along the *z* direction, which can be expressed as *F*_A0_(*z* − Δ*h*) = *F*_B0_(*z*). With this consideration, we derived the net buckling Δ*h* = 0.35 Å as shown in Extended Data Fig. 5d. Nevertheless, as the derived net buckling (Δ*h* = 0.35 Å) is close to the raw buckling (Δ*h* = 0.40 Å) that obtained without background-force subtraction, and the difference between them fade away when reducing the buckling to zero at the domain wall, we still use the raw buckling Δ*h* by simply calculating the height difference of the turning points of the Δ*f*(*z*) spectra to depict the wall thickness in Fig. 4.

### Domain manipulation

**Extended Data Fig. 7:**
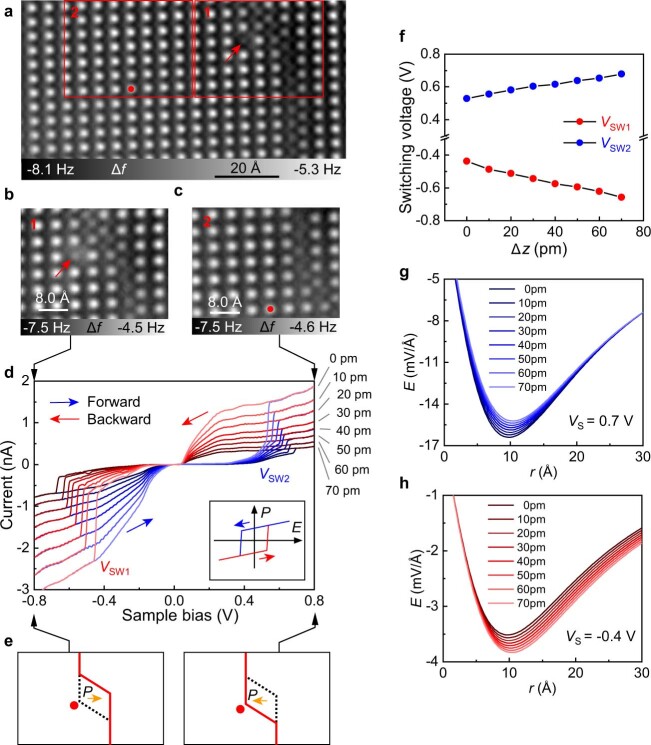
Extra data of the ferroelectric domain reversal. **a**, AFM image of a bent 180° head-to-head domain wall. **b**,**c**, AFM images acquired in the right (**b**) and left (**c**) red rectangle in (**a**) during the manipulation. Red dots show the tip position for the domain manipulation. The red arrow labels an atomic defect. **d**, Tunnelling current during the forward (blue series curves) and backward (red series curves) bias sweeping at different tip-sample distance (Δ*z* = 0 pm to 70 pm). The inset hysteresis loop schematically shows how the polarization of BP-Bi in the middle part is switched. **e**, Schematics show the position of the domain wall after the forward bias sweeping (right panel) and the backward bias sweeping (left panel). **f**, Extracted tip height-dependent switching voltages on the positive bias side (*V*_SW2_) and negative bias side (*V*_SW1_) from (**d**). **g**,**h**, Calculations of the tip height-dependent electric field evolution on the positive bias side (**g**) and negative bias side (**h**). Tip height *z* = −210 pm (**a**), −230 pm (**b**,**c**), and the initial tip height (Δ*z* = 0) in (**d**) is −50 pm, relative to the height determined by the setpoint *V* = 100 mV, *I* = 10 pA above normal Bi surface.

**Extended Data Fig. 8:**
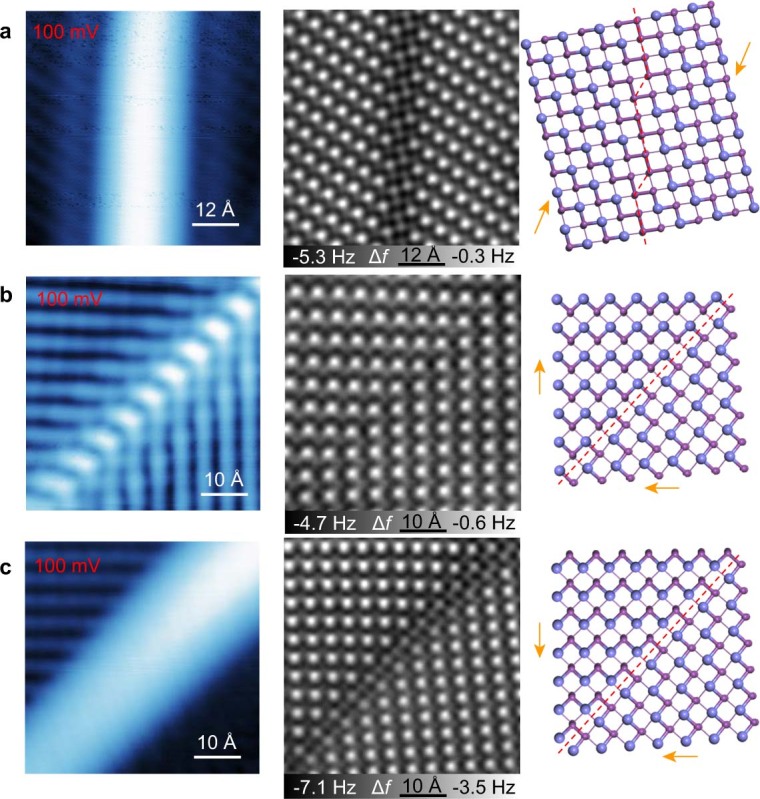
STM and AFM images of other types of domain walls. **a**–**c**, STM images (left panel), AFM images (middle panel) and corresponding atomic models (right panel) of the 54° inclined 180° head-to-head domain wall (**a**), 90° head-to-tail domain wall (**b**) and 90° head-to-head domain wall (**c**). Orange arrows denote the directions of polarization. Setpoints of the STM images in (**a**–**c**) are *V* = 100 mV, *I* = 10 pA (**a**), *V* = 100 mV, *I* = 30 pA (**b**), *V* = 100 mV, *I* = 50 pA (**c**). Tip heights to measure the AFM images in (**a**–**c**) are *z* = −250 pm (**a**), −250 pm (**b**), −200 pm (**c**), relative to the height determined by the setpoint *V* = 100 mV, *I* = 10 pA above normal Bi surface.

The polarization switching at another 180° head-to-head domain wall is shown in the Extended Data Fig. 7. Similar to the one in Fig. 3, the ferroelectric hysteresis during the electric field manipulation is distinguishable by the current variation at various tip-sample distances. But we note the switching voltage (*V*_SW_) changes from wall to wall because of the localized strain and defect, which is also the reason that causes the hysteresis loop a lateral shift so that deviates from the symmetric schematic in the inset of Fig. 3d (ref. ^56^).

To evaluate the electric field below the tip, we did the calculation in the prolate spheroidal coordinates (*η*, *ξ*). By treating the tip surface as a hyperboloid *η*_t_ and the graphite surface as an infinite metal plane *η* = 0, the potential in the tunnelling gap reads:

$${\psi }\left({\eta },{\xi }\right)={\Phi }_{{\rm{S}}}\left(1-\frac{1}{K}{\rm{ln}}\frac{1+\eta }{1-\eta }\right),$$

where sample potential *Φ*_S_ = *V*_S_ − *V*_CPD_ and

$$K=\mathrm{ln}\frac{1+{\eta }_{{\rm{t}}}}{1-{\eta }_{{\rm{t}}}}.$$

Transformation to the Cartesian coordinates (*r*, *z*) gives rise to the potential distribution *Φ*(*r*, *z*) and the derivative in-plane electric field *E*(*r*) at the BP-Bi plane (Fig. 3f,g).

In the meantime, the tip-height-dependent electric field at both switch sides can be calculated. As shown in Extended Data Fig. 7g,h, the electric field intensity (absolute value) below the tip decreases with the tip lifting at the positive bias side (*V*_S_ = 0.7 V), but increases at the negative bias side (*V*_S_ = −0.4 V). This suggests the electric field intensity is mainly dominated by the tip-sample distance elongation at the positive side, while by the decline of *V*_CPD_ at the negative side, which explains the upwards shift of *V*_SW0_, *V*_SW2_ and downwards shift of *V*_SW_, *V*_SW1_ when increasing the tip-sample distance (Fig. 3d,e and Extended Data Fig. 7f).

### Ferroelectric phase transition

**Extended Data Fig. 9:**
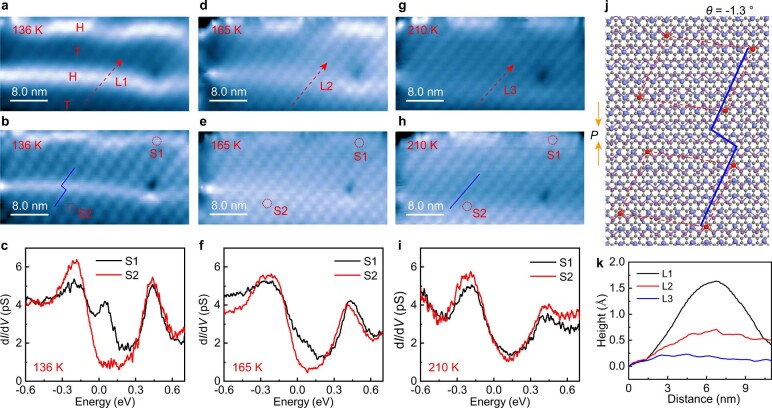
Temperature dependent phase transition. **a**–**i**, At the same area (marked by the defect in the right side of the scanning window), STM images above (**a**,**d**,**g**) and below (**b**,**e**,**h**) the Fermi level are measured at 136 K (**a**,**b**), 165 K (**d**,**e**) and 210 K (**g**,**h**). As labelled in (**b**,**e**,**h**), the d*I*/d*V* spectra at corresponding three temperatures 136 K (**c**), 165 K (**f**) and 210 K (**i**) are acquired above the 180° head-to-head domain wall (red) and a normal place (black). **j**, The structure model of the moiré pattern in this measured area shows how the 180° head-to-head domain wall induce a shift in the moiré superlattice (highlighted by the blue line). Only the top two layers of Bi atoms are included in the model for clarity. **k**, Line profile along the red dashed arrows in (**a**,**d**,**g**). Setpoint: *V* = 100 mV, *I* = 100 pA (**a**,**d**,**g**); *V* = −100 mV, *I* = 100 pA (**b**,**e**,**h**); *V* = −300 mV, *I* = 200 pA (**c**,**f**,**i**).

At higher temperatures, it is challenging to perform atomic force spectroscopy to extract the ferroelectric distortion directly. But the small atomic distortion can be magnified by the moiré pattern so that it is distinguishable by the STM topographic measurement directly. Particularly, the drastic buckling reversal at the 180° head-to-head domain wall contribute to a lateral shift of the moiré superlattice. This yields a kink of the moiré lattice crossing the wall. Extended Data Fig. 9 shows the domain wall we measured at a series of different temperatures in the same area. At low temperatures, the kink produced by the 180° head-to-head domain wall is easy to find (blue line), but it smears at 165 K and disappears at 210 K. Apart from the STM topography measurement, we did the d*I*/d*V* measurement at the domain wall, and found the peak of the in-gap state also shows similar behaviour, that is, the peak weakened with the rise of the temperature and disappeared at 210 K. The gradual changes of the kink and spectra not only exclude the tip-induced domain manipulation that could move the domain wall away by the electric field, but also derive a Curie temperature of about 210 K with a possible second-order phase transition.

### Calculation of the order parameter profile at the 180° head-to-head and tail-to-tail domain wall

We use a three-dimensional (3D) counterpart that contains multilayers of 180° head-to-head or tail-to-tail domain walls along the *z* direction to inspect the development of the domain profile. The equivalent 3D model with an adjusted interlayer distance has the merits of involving the screening effect of the substrate and simplifying the calculation to be one-dimensional. To satisfy the free energy minimization of the ferroelectrics with second-order phase transition, we have^36,43,44^

$$\alpha P+\beta {P}^{3}=k\frac{{\partial }^{2}P}{{\partial x}^{2}}-\frac{\partial \phi }{\partial x},$$

where *x* is the axis perpendicular to the domain wall plane; *α*, *β*, *k* have the same definitions as those in the main text and *β* > 0, *α* = *α*_0_(*T* − *T*_C_) with the constant *α*_0_ > 0. In the meantime, the electric potential *φ* and polarization *P* across the domain wall (along the *x* axis) fulfil the Poisson equation

$$\varepsilon \frac{{\partial }^{2}\phi }{\partial {x}^{2}}=\frac{\partial P}{\partial x}-\rho ,$$

where the *ε* is the permittivity, *ρ* is the charge density that mainly contributed by the band edges of BP-Bi. According to the density of states (DOS) features reflected by the d*I*/d*V* curve and the QPI measurement in the Extended Data Fig. 4, we approach the carrier concentration by using the parabolic band edge at the CBM and a non-parabolic mode at the VBM with the fitted parameters. At *T* = 4.3 K under the 2D limit, they can be approximated as

$${n}_{{\rm{e}}}=\frac{2{m}_{{\rm{C}}}{E}_{{\rm{n}}}}{\pi {\hbar }^{2}},{E}_{{\rm{n}}}={E}_{{\rm{F}}}-{E}_{{\rm{C}}}+\phi e,$$

$${n}_{{\rm{h}}}=\frac{2{m}_{{\rm{V}}}}{\pi {\hbar }^{2}}\left({E}_{{\rm{r}}}+\frac{a}{2}{E}_{{\rm{r}}}^{2}\right),{E}_{{\rm{r}}}={E}_{{\rm{V}}}-{E}_{{\rm{F}}}-\phi e,$$

Here, $\hbar$ is the reduced Planck’s constant, *E*_F_ is the Fermi energy level, *e* is the absolute value of the electron charge, *m*_C_ (*m*_V_) and *E*_C_ (*E*_V_) is the effective-mass and the energy of the conduction (valence) band edge, respectively. The solving of the differential equations above produces the conjugated results at the head-to-head and tail-to-tail wall (blue curves in Fig. 4i–l).

When considering the Coulomb screening of the ferroelectric dipole, we calculate the Curie temperature at different screening length or charge concentration to include the screening term^13,57^. Thus, we rewrite the coefficient *α* as *α*_0_(*T* − *T*_C_(*ρ*)). From the results of the red dashed curved in Fig. 4k, substantial wall broadening can be observed because of the weakening of the spontaneous polarization near the wall. Further consideration of the Fermi energy at different buckling level (Δ*h*) is conducted by introducing an extra carrier-concentration term *ρ*′(Δ*h*), which is approximated linearly by referring to the DFT calculations (Extended Data Fig. 1c).

## Online content

Any methods, additional references, Nature Portfolio reporting summaries, source data, extended data, supplementary information, acknowledgements, peer review information; details of author contributions and competing interests; and statements of data and code availability are available at 10.1038/s41586-023-05848-5.

## Supplementary information

Supplementary Information Supplementary Sections 1–9, Figs. 1–9 and Tables 1 and 2.

## Figures and Tables

**Extended Data Fig. 6:**
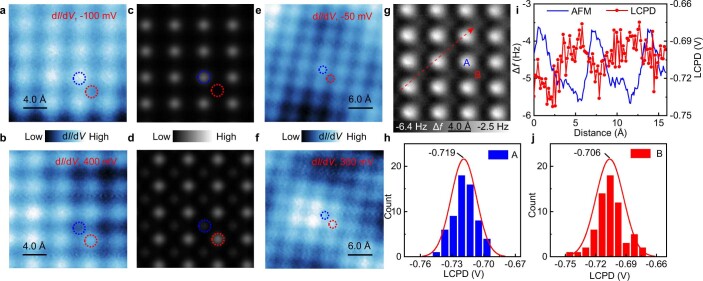
Extra data of electron redistribution by STM and KPFM measurement. **a**,**b**, At the identical area of Fig. 2a in the main text, d*I*/d*V* maps at other energy show the same DOS reversal between the valence band (**a**) and conduction band (**b**). **c**,**d**, The calculated charge density distribution of the valence band (**c**) and conduction band (**d**) of *p*_z_ at the Γ point. **e**,**f**, d*I*/d*V* maps of the valence band (**e**) and conduction band (**f**) measured by a metallic tip apex. Large-scale bumps and depressions come from the moiré pattern. Blue dotted circles and red dotted circles represent the A and B sublattice, respectively. **g**, AFM image of a normal area away from the domain walls. **h**,**j**, Histograms of LCPD measured in a 1.5 × 1.5 Å^2^ square (8 × 8 grid) centred above the A atom (**h**) and B atom (**j**) in (**g**). The difference of the two Gauss peaks is 13 mV. **i**, AFM profile (blue) and KPFM measurement (red) along the same trajectory marked by the red dashed arrow in (**g**). Tip height *z* = −150 pm (**a**,**b**,**e**), −200 pm (**f**), −230 pm (**g**), −80 pm (**h**–**j**), relative to the height determined by the setpoint *V* = 100 mV, *I* = 10 pA above normal Bi surface.

## Data Availability

The main data supporting the finding of this work are available within this paper. Extra data are available upon reasonable request from the corresponding authors.

## Code availability

The computer code used for numerical calculation and data processing are available from the corresponding authors upon reasonable request.
