# Supplementary material for: Two-dimensional ferroelectricity in a single-element bismuth monolayer
Source: Nature. 2023 Apr 5;617(7959):67–72. doi: 10.1038/s41586-023-05848-5 (PMC10156600; doi:10.1038/s41586-023-05848-5)
Supplement: Supplementary file 1 — Supplementary Sections 1–9, Figs. 1–9 and Tables 1 and 2. [file 41586_2023_5848_MOESM1_ESM.pdf]

nature portfolio

https://doi.org/10.1038/s41586-023-05848-5

Supplementary information

Two-dimensional ferroelectricity in a single-element bismuth monolayer

In the format provided by the
authors and unedited

Supplementary Information for

Two-dimensional ferroelectricity in a single-element bismuth monolayer

Jian Gou[1*], Hua Bai[2,3], Xuanlin Zhang[2], Yu Li Huang[4], Sisheng Duan[1], A. Ariando[1], Shengyuan A. Yang[5], Lan Chen[6,7*], Yunhao Lu[2*], Andrew Thye Shen Wee[1,8*]

[1]Department of Physics, National University of Singapore, 2 Science Drive 3, Singapore 117542, Singapore
[2]Zhejiang Province Key Laboratory of Quantum Technology and Device, School of Physics, State Key Laboratory of Silicon Materials, School of Materials Science and Engineering, Zhejiang University, Hangzhou 310027, China
[3]Department of Physics, Faculty of Science, Kunming University of Science and Technology, Kunming, 650500, China
[4]Joint School of National University of Singapore and Tianjin University, International Campus of Tianjin University, Fuzhou, 350207, China
[5]Research Laboratory for Quantum Materials, Singapore University of Technology and Design, Singapore 487372, Singapore
[6]Institute of Physics, Chinese Academy of Sciences, Beijing 100190, China
[7]School of Physics, University of Chinese Academy of Sciences, Beijing 100049, China
[8]Centre for Advanced 2D Materials (CA2DM) and Graphene Research Centre (GRC), National University of Singapore, Singapore 117546, Singapore

[*]e-mail: phygouj@nus.edu.sg; lchen@iphy.ac.cn; luyh@zju.edu.cn; phyweets@nus.edu.sg

This file includes:

Section 1. Work function measurements and band evolutions at tail-to-tail domain wall.
Section 2. In-gap states at head-to-head domain wall.
Section 3. Orientation dependence of band bending.
Section 4. Domain wall stability.
Section 5. Band bending at BP-Bi edges.
Section 6. Continuous movement of charged domain walls.
Section 7. Ferroelectric switching near other types of domain walls.
Section 8. Effects of substrate and defect.
Section 9. Tip-height dependent switching voltages.
Fig. S1 Work function measurement of two different tail-to-tail domain walls.
Fig. S2 Electronic structure of the localized in-gap states.
Fig. S3 Orientation dependence of band bending at different domain walls.
Fig. S4 Band bending measurement at BP-Bi edges.
Fig. S5 Continuous manipulation of a domain wall in BP-Bi island.
Fig. S6 STM image of the BP-Bi island where the polarization switching in Fig. 3 is performed.
Fig. S7 Domain manipulations on other types of domain walls.
Fig. S8 Defects and charge transfer in BP-Bi.
Fig. S9 AFM image of BP-Bi measured at 210 K.
Table S1 DFT derived local relative formation energy at different domain walls in BP-Bi.
Table S2 Calculated local ICOHP at different domain walls for BP-Bi and SnTe.

1. Work function measurements and band evolutions at tail-to-tail domain wall

As shown in Fig. S1 and Fig. S3, both *I-Z* spectra and KPFM measurements across different types of domain walls were performed. The two methods within the measurement accuracy show the same bending tendency. This can be understood by the following analysis: the local tunneling barrier height derived by *I-Z* spectra contains the work functions of both sample and tip, and the KPFM measurement gives the local potential difference (LCPD) between sample and tip. Since LCPD can be treated as the work function difference here, the measured barrier height and LCPD across the domain wall with a certain tip work function should produce the same band bending of the walls.

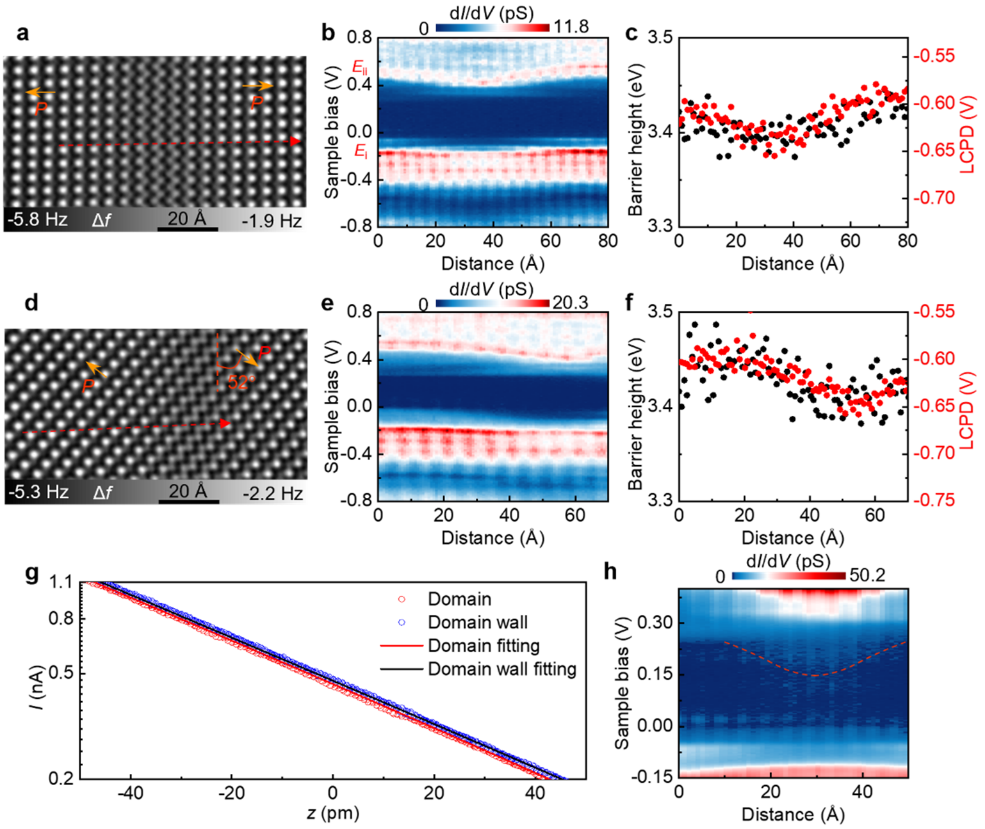

Fig. S1 | Work function measurement of two different tail-to-tail domain walls. a,d, AFM images of a 90° inclined 180° tail-to-tail domain wall (**a**) and a 52° inclined 180° tail-to-tail domain wall (**d**). **b,c,** d*I*/d*V* line map (**b**) and work function measurement (**c**) along the red dashed lines in (**a**). **e,f,** d*I*/d*V* line map (**e**) and work function measurement (**f**) along the red dashed lines in (**d**). Barrier height and LCPD were extracted by fitting the slope of ln *I*(*Z*) and the parabolic maximum of Δ*f*-*V* spectra, respectively. **g,** Two typical *I-Z* spectra taken at the domain (red circles) and domain wall (blue circles) in (**a**). **h,** Close d*I*/d*V* line map along the red dashed lines in (**a**) within only a distance of 50 Å from the beginning (initial setpoint: *V* = -0.15 V, *I* = 0.4 nA). Tip height z = -240 pm (**a,d**), -50 pm (**b**), -50 pm for LCPD and 20 pm for barrier height in (**c,g**), -70 pm (**e**), -70 pm for LCPD and 30 pm for barrier height in (**f**), relative to the height determined by the setpoint *V* = 100 mV, *I* = 10 pA above normal BP-Bi surface.

According to the DFT calculations on the buckling dependence (Extended Data Fig .1c), besides the increase of work function, the calculations depict a decrease of the band gap near the tail-to-tail domain wall at the same time. In the experiment, we performed the STS measurements within a narrow energy range around the gap at the wall, as shown in Fig. S1h. The expected smaller band gap at the domain wall can be unambiguously identified, where the weak conduction band edge is at around 0.15 V (red dashed curve in Fig. S1h). Besides, the band movement related to the band gap narrowing can also be confirmed by the VB and CB movement towards each other (the interval reduction of about 0.1 eV between E_{ii} and E_i) (Fig. 4d and Fig. S1b).

2. In-gap states at head-to-head domain wall

The in-gap states at head-to-head domain wall in Fig. 1h in the main text can be explained to be a localized bound state that produced by the bound charge induced electric field. Similar localized state at the 180° ferroelectric domain wall can also be found in the experimental studies of SnSe [1] and theoretical calculations of GeTe[2]. The localization at the one-dimensional (1D) head-to-head domain wall in our case generates a 1D electron gas whose 1D parabolic dispersion can be characterized by the perfect fitting to a typical 1D parabolic DOS formula $D \propto 1/\sqrt{E - E_0}$ (Fig. 1h).

To confirm the 1D localized state, we calculated the electronic band structure of a freestanding supercell with 180° head-to-head domain wall. It is obvious the electronic structure projected to the head-to-head domain wall shows a 1D parabolic band structure near the Fermi surface (Fig. S2, red circles). In contrast, the absence of the bound charge at the 90° head-to-tail domain wall lacks the localized electronic states (Fig. S3d).

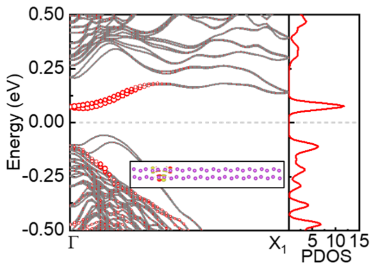

Fig. S2 | Electronic structure of the localized in-gap states. Band structure (left panel) of the supercell (gray lines) with the red circles represent the contributions of 180° head-to-head domain wall. Right panel shows the partial density of states (PDOS) projected to the wall. Charge density distribution of electronic states with energy near 0-0.1 eV is shown in the inset.

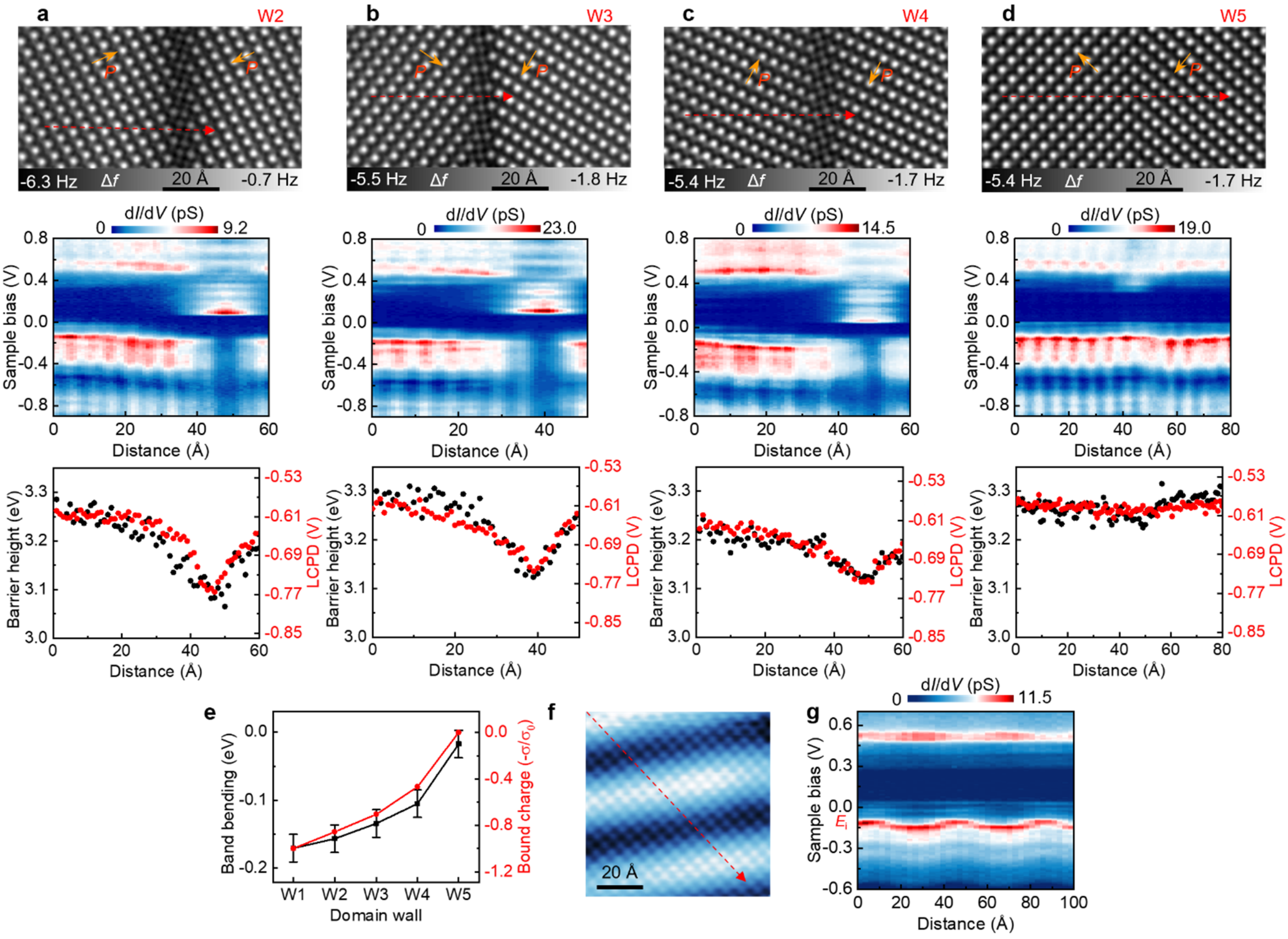

Fig. S3 | Orientation dependence of band bending at different domain walls. a-d, AFM images (top panel), dI/dV line maps (middle panel) and surface potential (bottom panel) measured at the 59° inclined 180° head-to-head domain wall (**a**), 90° head-to-head domain wall (**b**), 28° inclined 180° head-to-head domain wall (**c**) and 90° head-to-tail domain wall (**d**). Barrier height and LCPD were extracted by fitting the slope of ln $I(Z)$ and the parabolic maximum of Δf -V spectra, respectively. Spectrum measurement was performed along the trajectories marked by the red dashed arrows at respective domain wall. **e**, Band bending measured in the experiment and the normalized theoretical bound charge density (σ_0 is the theoretical bound charge of 90° inclined 180° head-to-head domain wall). Band bending in each case was derived by computing the difference between LCPD of the first point and LCPD measured at the domain wall (W1: Fig. 4f, W2-W5: (**a-d**)). Error bars represent the standard deviation from multiple measurement plus half of the potential difference between A and B sublattice. **f,** STM image of a typical stripe-like moiré pattern (V = -0.1 V, I = 50 pA). **g**, dI/dV line map along the red dashed line in (**f**) show a strong modulation of E_i by the moiré stripes. Tip height for top panel of (**a-d**) z = -250 pm (**a**), -230 pm (**b-d**); Tip height for middle panel of (**a-d**) and the LCPD measurement in bottom panel of (**a-d**) z = 30 pm (**a**), -50 pm (**b**), -40 pm (**c**,**d**); Tip height for the barrier height measurement in bottom panel of (**a-d**) z = -30 pm (**a**), -90 pm (**b**), -70 pm (**c**,**d**); Tip height z = -50 (**g**). z = 0 pm is determined by the setpoint V = 100 mV, I = 10 pA above normal BP-Bi surface.

3. Orientation dependence of band bending

To find out the relationship between the incline angle and band bending (bound charge density), we performed both *I-Z* spectra and KPFM to measure the work function changes at several kinds of domain walls, which are 59° inclined 180° head-to-head domain wall (W2, Fig. S3a), 90° head-to-head domain wall (W3, Fig. S3b), 28° inclined 180° head-to-head domain wall (W4, Fig. S3c) and 90° head-to-tail domain wall (W5, Fig. S3d). The work function variation measured by *I-Z* spectra (barrier height) and KPFM (LCPD) reveal the same results for each domain wall. It is noted that the domain walls with small incline angle appear parallel to the stripe-like moiré pattern (Extended Data Fig. 2a,d), here we use the *I-Z* spectra instead of the E_i peak to prevent any disturbance caused by the moiré-induced band modulation (Fig. S3f, S3g).

Since LCPD in the experiments has a relatively higher accuracy, we derive the band bending in each case by calculating the difference between LCPD at the wall and at the domain (the first measuring point). Involving Fig. 4f in the main text together (as W1), the orientation-dependent band bending shows a gradual decrease from W1 to W5 (Fig. S3e). In theory, the bound charge density can be ploted via $\sigma = (\boldsymbol{P_1} - \boldsymbol{P_2}) \cdot \boldsymbol{n}$, where $\boldsymbol{P}_1$ and $\boldsymbol{P}_2$ are the polarization vectors in the neighboring domains and $\boldsymbol{n}$ is the unit vector perpendicular to the wall. Thus, for the head-to-head domain wall, this can be considered equivalent to $\sigma = 2P_s \times \cos(90°- \rho)$ (inclinde angle ρ = 90°, 59°, 45° and 28° for W1-W4), while for the head-to-tail domain wall σ is calculated to be zero (W5). From the comparision between the band bending measurements and the theoretical bound charge calculations (Fig. S3e), the orientation dependence of the band bending can be clearly identified at different domain walls, which supports the macroscopic spontaneous polarization in BP-Bi.

4. Domain wall stability

For the stability of different domain walls in BP-Bi, we calculated the 180° domain walls and 90° domain walls to investigate the basic atomic structure and relative formation energy. As shown in Table S1, all the 90° domain walls (77.0 meV/Å and 93.5 meV/Å) exhibit a higher energy than 180° domain wall (29.1 meV/Å). The analysis on the bond strength by computing the integrated crystal orbital Hamilton population (ICOHP) also reveals a weaker bonding at the 90° domain walls even though it is neutral (Table S2), suggesting that 180° charged domain wall in BP-Bi is more favorable than the 90° domain wall. For comparison, we also checked ICOHP of 180° charged head-to-head and 90° neutral head-to-tail domain wall in SnTe. As shown in Table S2, the 90° neutral head-to-tail domain wall shows a stronger bonding than the 180° charged head-to-head domain wall, coinciding with the frequently observed 90° neutral head-to-tail domain wall in SnTe[3].

Why the stability of 90° neutral domain wall and 180° charged domain wall is totally opposite for BP-Bi and SnTe? We noticed the relative changes of ICOHP for 90° neutral head-to-tail domain wall in BP-Bi is larger than that in SnTe. Considering that BP-Bi (a = 4.79 Å, b = 4.51 Å) has a stronger lattice anisotropy than SnTe (a = 4.58 Å, b = 4.55 Å)[4], we infer that strain dominates the stability of domain wall in BP-Bi rather than the electrostatic energy in

SnTe. This is understandable since BP-Bi has a smaller bandgap (0.26 eV) than SnTe (1.6 eV), and BP-Bi is even heavily p-type doped in our measurement. The dense free charge carriers in BP-Bi largely compensate the bound charge to make the charged 180° domain walls more stable or the electrostatic energy not dominated.

Table S1 | DFT derived local relative formation energy at different domain walls in BP-Bi.

Domain wall Types		E_F (meV/Å)
180°	Head-to-head & tail-to-tail	29.1
90°	Head-to-tail	77.0
90°	Head-to-head & tail-to-tail	93.5

Table S2 | Calculated local ICOHP at different domain walls for BP-Bi and SnTe.

Domain Wall Types		ICOHP	Relative changes (refer to monodomain)
BP-Bi	Monodomain	8.369	--
	180° head-to-head	8.428	0.7%
	90° head-to-tail	8.194	2.1%
SnTe	Monodomain	8.347	--
	180° head-to-head	7.857	5.9%
	90° head-to-tail	8.224	1.5%

5. Band bending at BP-Bi edges

It is noticeable that since the buckling near the edge is preserved, there should be a difference in the band movement even both edges are regularly reconstructed. However, depending on both STM (Extended Data Fig. 5e,g, Fig. S4a,d) and AFM images (Extended Data Fig. 5f,h), we found only the 'tail' edge (with polarization away from the brim) is regularly reconstructed, while the 'head' edge (with polarization towards the brim) is irregular. There are extra adatoms adsorbed at the 'tail' edges with four times period of the lattice constant of BP-Bi. At the 'head' edge, the adatoms distributed irregularly at the brim (Fig. S4d, Extended Data Fig. 5e). The different amount of adatoms with distinct adsorption manner bring in different local doping levels, extra dangling bonds, and local strain distributions, which should impose important

influence on the local band arrangement. Therefore, it is difficult to distinguish what the measured differences between the two kinds of edges come from. Figure S4 shows the d*I*/d*V* measurements at both edges with trajectories perpendicular and parallel to the edges, The results exhibit a metallic and heavily electron doped edges with downward band bending for both cases. The in-gap states in the d*I*/d*V* spectra also indicate the existence of extra electrons contributed by the adatoms at both edges.

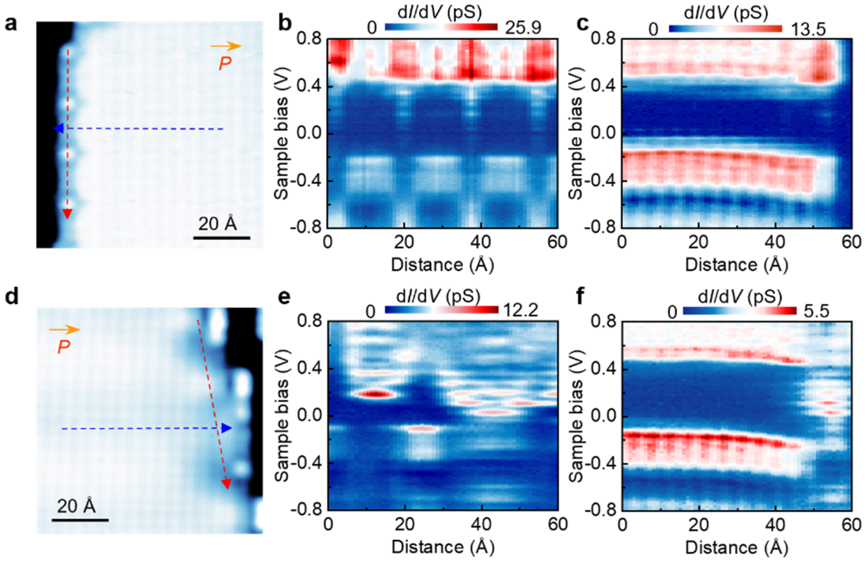

Fig. S4 | Band bending measurement at BP-Bi edges. a,**d**, STM images of the BP-Bi edge with polarization away from (**a**) and towards (**d**) the brim. **b**,**c**, d*I*/d*V* spectra measured along the red dashed line (**b**) and blue dashed line (**c**) in (**a**). **e**,**f**, d*I*/d*V* spectra measured along the red dashed line (**e**) and blue dashed line (**f**) in (**d**). Setpoint V = -0.8 V, I = 10 pA (**a**,**d**); V = -0.8 V, I = 500 pA (**b**). Tip height z = -40 pm (**c**), -50 pm (**e**), -30 pm (**f**) relative to the height determined by the setpoint V = 100 mV, I = 10 pA above normal Bi surface.

6. Continuous movement of charged domain walls

In principle, an unpinned domain wall can be moved to any location, the bias voltage that switches a regular ferroelectric domain wall at a fixed tip position is not that repeatable. In our experiment, we find some domain walls start or end at an irregular edge or the endpoint of the second BP-Bi layer (Extended data Fig. 2a), suggesting these domain walls are pinned. At this situation, the switching bias voltage can be highly repeated in different measurements. On the other hand, the domain wall with less pinning force can easily be dragged through a relatively long distance. As shown in Fig. S5a-d, the head-to-head domain wall in the BP-Bi island can be dragged from the right side to the left side by STM tip. However, as the wall is significantly longer than the effective range of the tip-induced electric field, and the pinning potential at each end of the wall is not equal, we found it was much easier to drag the wall by manipulating one end at a time, separately (Fig. S5b,c).

Moreover, we also performed the voltage pulse to switch the polarization. As shown in Fig.

S5d,e, a voltage pulse with tens of milliseconds was found to be capable to realize the ferroelectric switching near the STM tip. However, the pulse with width less than 1 ms cannot induce effective movement.

The domain walls are stable after withdrawing the tip bias. As an example in Fig. 3c of the main text, the AFM measurement was carried out with zero sample bias after the manipulation. Meanwhile, the stability can also be confirmed by the STM measurement in Fig. S5 after the voltage ramp or pulse, in which the scanning does not change the position of the domain wall when tip moves away from the wall. Therefore, the polarization manipulation for BP-Bi is a nonvolatile switching.

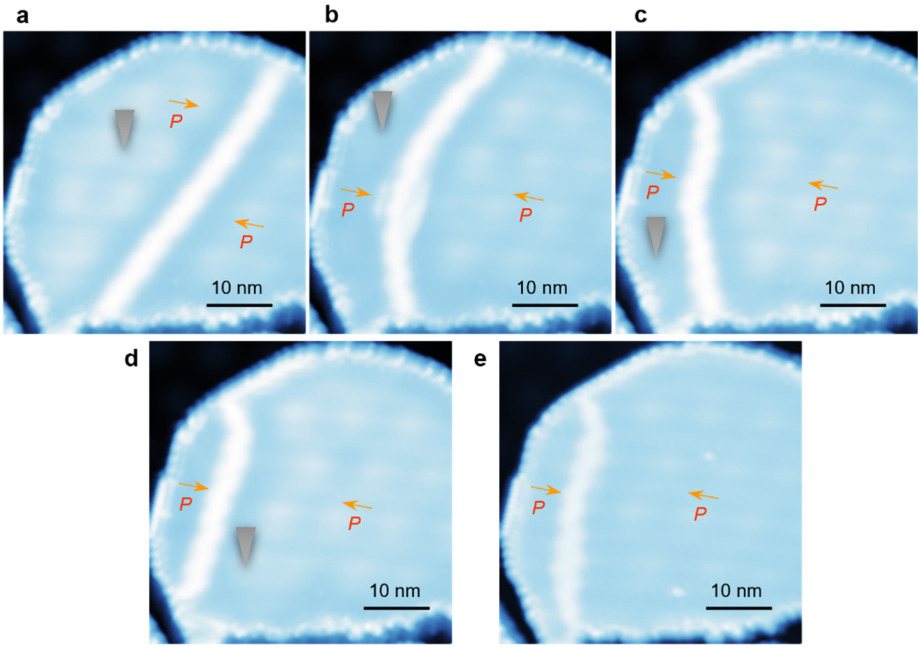

Fig. S5 | Continuous manipulation of a domain wall in BP-Bi island. a-d, STM images (V = 200 mV, I = 4 pA) show how the head-to-head domain wall was dragged from the right side (**a**) step-by-step (**b**,**c**) to the left side (**d**) of the BP-Bi. As marked by the gray triangles in (**a-d**), domain wall manipulation was performed by putting the tip aside the domain wall and gradually ramping the setpoint to V = 4.0 V, I = 10 pA (**a-c**) or applying a voltage pulse of 3.0 V, 20ms at corresponding setpoint (**d**). Then the setpoint was recovered to check the position after the manipulation, the result of each manipulation was shown in the next STM image in (**b-e**). Setpoint in (**e**): V = 400 mV, I = 4 pA.

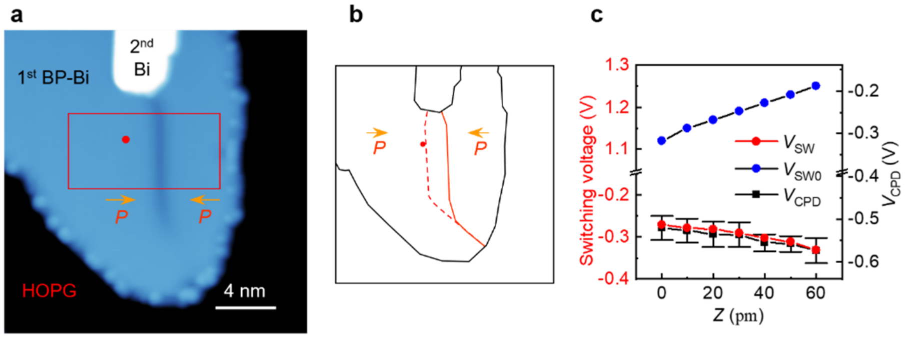

Fig. S6 | STM image of the BP-Bi island where the polarization switching in Fig. 3 is performed (a). Red solid line and red dashed line in (**b**) schematically show the locations of domain wall before and after the manipulation. The red rectangle in (**a**) marks the area where the AFM image in Fig. 3a was measured and the red dots indicate the location of tip during the manipulation (setpoint: V = -1V, I = 10pA). **c**, The copy of Fig. 3e in the main text with the switching voltage V_{SW0} at positive bias side added.

7. Ferroelectric switching near other types of domain walls

We systematically investigated the manipulation of the 180° tail-to-tail domain walls, and found they were able to be manipulated with a higher bias voltage. As shown in Fig. S7a, a 180° tail-to-tail domain wall is originally pinned to the right side by a defect (red arrow), leading to the depression at negative sample bias due to less conductivity at the wall. With a higher bias voltage (-3.0 V), a jump of the tip height as well as the transformation of tail-to-tail domain wall to a normal domain area can be observed (Fig. S7a, top panel), which indicates the tail-to-tail domain wall is ejected away from the tip and moves to the left side of the scanning window. Similarly, the domain wall will jump back (recover) as the tip moves to the left side of the defect (Fig. S7a, bottom panel). The manipulation of the tail-to-tail domain wall was also examined continuously by the *I*-*V* spectra acquired at the wall (Fig. S7c). A gradual decrease in (negative) sample bias leads to a current jump at V_{SW3} that reflects the ejection of the domain wall away from the tip side, while the recovery of current curve at V_{SW4} indicates the pulling back of the wall when increasing the bias voltage. The two switching voltages (V_{SW3} and V_{SW4}) similarly exhibits the tip height dependence as that in the head-to-head domain wall manipulations (Fig. 3 and Extended Data Fig. 7f). The interval between the two switching voltages (e.g., V_{SW4} - V_{SW3}) increases with the tip rise in order to reach the same critical electric field. The shift of the switching loop can be attributed to the pinning of the defect[5].

In the meanwhile, we noted the manipulation of tail-to-tail domain wall has the same bias voltage polarity as head-to-head walls. This may be understood that the tail-to-tail domain wall with buckling reconstruction has a higher work function, which is equivalent to positive bound charges at the wall rather than negative bound charges at an ordinary tail-to-tail domain wall.

Except the 180° tail-to-tail domain wall, other 180° walls along a random direction can also be manipulated. For example, the 50° inclined 180° tail-to-tail domain wall was pinned by the stripe-like moiré pattern and can be manipulated to move in between two moiré periods by

forward and backward scanning at -3.8 V (Fig. S7e). The 180° tail-to-tail and head-to-head domain walls with randomly distributed incline angle can be manipulated to disappear, thereby forming a single domain (Fig. S7f,g). However, the 90° head-to-head and 90° head-to-tail domain walls were not successfully manipulated in the bias range of ±5 V. This can be explained by the DFT calculations: the migration energies of 90° head-to-head and 90° head-to-tail domain walls are an order of magnitude higher than the 180° head-to-head domain wall (Fig. S7h-k).

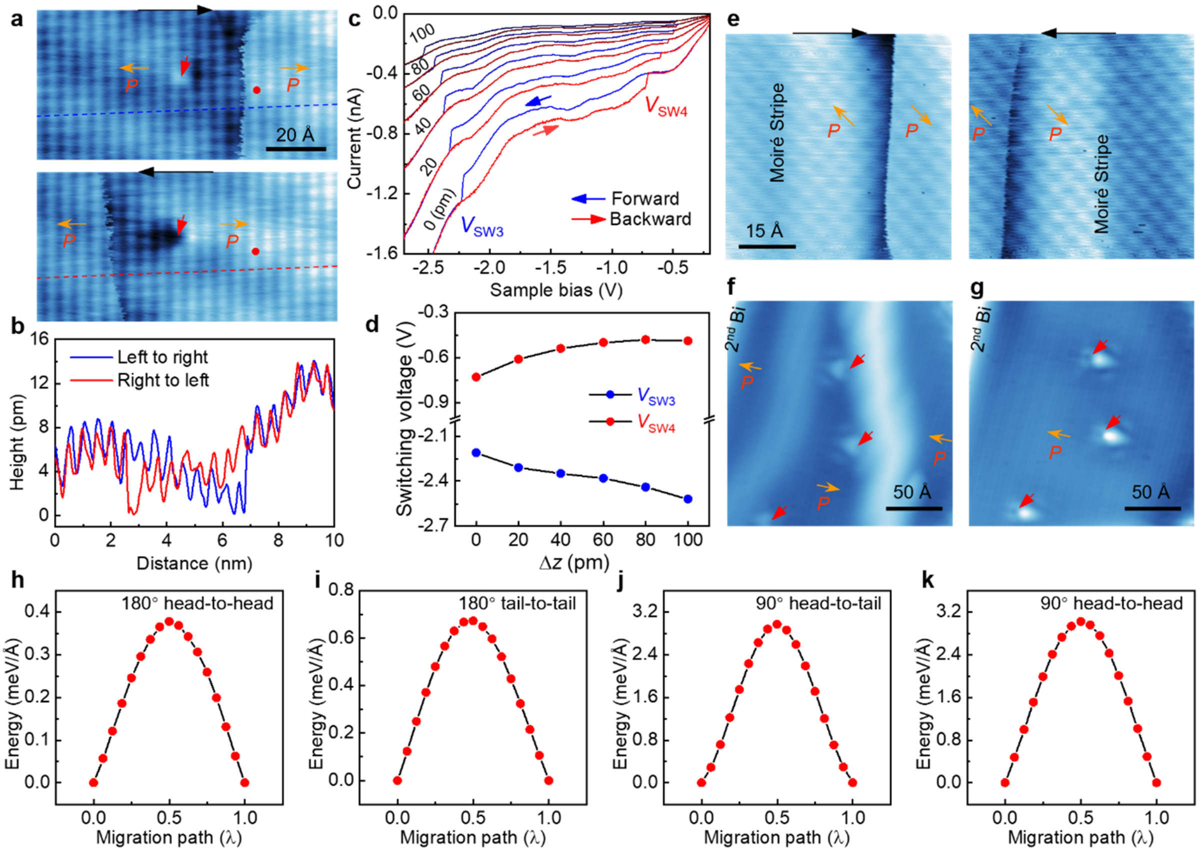

Fig. S7 | Domain manipulations on other types of domain walls. **a**, STM images of the 180° tail-to-tail domain wall under a high bias voltage (-3.0 V, 500 pA). Forward channel (from left to right) and backward scanning channel (from right to left) are shown in the top panel and bottom panel, respectively. Red arrows mark the same defect under opposite polarization. **b**, line profile along the dashed line in the top panel (blue) and the bottom panel (red) in (**a**) present the contrast jump clearly. The contrast jumping indicates a reject of the 180° tail-to-tail domain wall away from the tip position. **c**, Tunnelling current during the forward (blue series curves) and backward (red series curves) bias sweeping at different tip-sample distance (Δz = 0 pm to 100 pm). Red circles in (**a**) mark the tip position to do the I-V spectra. **d**, Extracted tip height-dependent switching voltages of the forward (V_{SW3}) and backward (V_{SW4}) bias sweeping in (**c**). **e**, STM images of a 50° inclined 180° tail-to-tail domain wall under a high bias voltage (-3.8 V, 100 pA). Forward channel (from left to right) and backward scanning channel (from right to left) are shown in the left panel and right panel, respectively. **f**,**g**, STM images (-0.4 V, 10 pA) of the same area before (**f**) and after (**g**) the manipulation by a high bias voltage (-2.6 V, 100 pA) scan. Three defects (highlighted by the red arrows) change their shape to reversed ones after the

domain's polarization is switched. **h**-**k**, Computed migration barriers for 180° head-to-head domain wall (**h**), 180° tail-to-tail domain wall (**i**), 90° head-to-tail domain wall (**j**) and 90° head-to-head domain wall (**k**). λ stands for half a unit cell along the orthogonal direction of the domain wall.

8. Effects of substrate and defect

We found the KPFM measurements in the experiment exhibited a higher surface potential on substrate (HOPG) than that on BP-Bi (Fig. S8a). This means even at a balanced state, there is a higher work function of HOPG than that of BP-Bi. Thus, we infer that electrons transfer from Bi to HOPG and result in the observed intrinsic p-type doping in the BP-Bi layer. Our followed calculations of Bi/Graphene superlattice (Extended Data Fig. 2c) with different Graphene layers underneath also shows the same charge transfer manners (Fig. S8b,c).

In the AFM measurements, the most common defect can be identified is Bi vacancy (Fig. S8d). We also performed DFT simulations on two intrinsic defects: Bi vacancy and Bi adatom (Fig. S8e,f). Their computed formation energies indicate the Bi vacancy (0.27 eV) is more stable than Bi adatom (1.03 eV), verifying the observed defect is Bi vacancy. Assuming the defect is 100% ionized and treated as background charge, the carrier density contributed by the Bi vacancy can be determined by counting the density of the defect directly in large-area STM images (Extended Data Fig. 4b is an example). Then, the charge carrier concentration contributed by defect can be estimated to be 2.3×10^{11} cm^{-2}. Accordingly, these charge carriers will enhance the Coulomb screening. On the other hand, the total background free holes of 7.6×10^{12} cm^{-2} that induced by charge transfer between Bi and substrate can be calculated from the fitted hole pocket and the energy shift of VBM (30 meV) in Extended Data Fig. 4f. Since the charge carrier density contributed by defects is quite small compared with carrier concentration caused by the Bi-substrate charge transfer, and the defect in fact is not 100% ionized at the measuring temperature (4.3 K), therefore we neglect the defect in our model to simplify the domain wall calculations.

Since HOPG substrate has a higher work function than BP-Bi and is semimetallic, the effects applied to BP-Bi contain two aspects that contribute to the screened Coulomb interaction: (i) the abundant charge carriers induced by the charge transfer between Bi and substrate; (ii) the image dipoles induced in the semimetallic graphite substrate. Charge carriers reduce the Thomas-Fermi screening length, and the image dipoles are oppositely orientated with the dipoles of BP-Bi. Both effects would strengthen the screened Coulomb interaction in BP-Bi and consequently broaden the domain wall width.

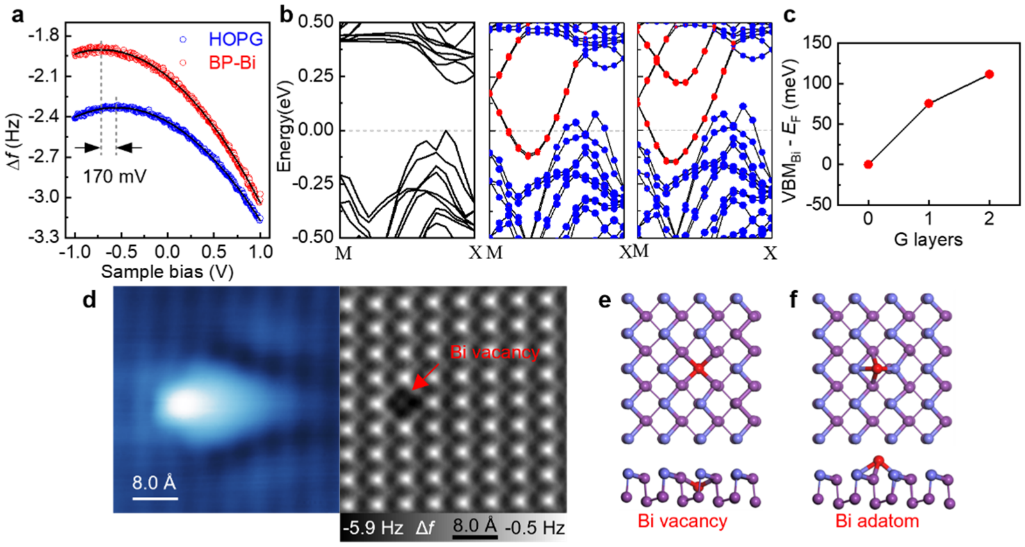

Fig. S8 | Defects and charge transfer in BP-Bi. a, KPFM measurements on BP-Bi (red circles) and nearby HOPG substrate (blue circles) with same tip. The fitted LCPD (black curves) from them indicate a higher surface potential on HOPG than that on BP-Bi. Tip height z = 150 pm on HOPG relative to the height determined by the setpoint V = 100 mV, I = 10 pA above HOPG, z = -50 pm on BP-Bi relative to the height determined by the setpoint V = 100 mV, I = 10 pA above normal BP-Bi surface. Both tip heights have same z shift (150 pm) relative to the turning point of $\Delta f(z)$ spectra measured on the topmost C atom or Bi atom. **b**, Band structure of Bi/Graphene superlattice (Extended Data Fig. 2c) focused on the VBM of Bi with the consideration of no substrate (left panel), single-layer graphene (middle panel) and two-layer graphene (right panel) underneath to inspect the effects of graphite substrate. The electronic bands of carbon and Bi are highlighted by red and blue circles, respectively. **c**, The VBM shift of Bi relative to the Fermi surface as a function of graphene layers shows the increase of hole doping caused by electron transfer. **d**, STM image (left panel, V = 0.37 V, I = 30 pA) and AFM image (right panel, z = -270 pm) of a Bi-vacancy defect. **e**,**f**, Top view (top panel) and side view (bottom panel) of the optimized Bi-vacancy (**e**) and Bi-adatom (**f**) defect.

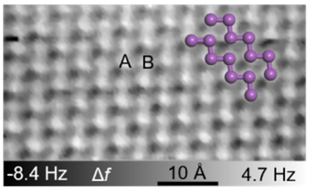

Fig. S9 | AFM image of BP-Bi measured at 210 K. The nearly equal contrast between A and B sublattice shows a nearly flat surface in BP-Bi, which indicates the evidence of a transition to a centrosymmetric parent phase at around 210 K. A buckling-free model with only top atoms are superimposed to highlight the atom positions.

9. Tip-height dependent switching voltages

The domain walls used for polarization switch are ordinarily pinned by a second layer BP-Bi (Fig. S6a) or a defect (Extended Data Fig. 7a), allowing us to observe the repeatable hysteresis loop in *I-V* spectra. As different pinning potential of these pinning effects cause a diverse lateral shift of hysteresis loops[5], along with the height dependent V_{CPD} of HOPG, different polarization switches produce different asymmetric characteristics.

Specifically, since V_{CPD} in the experiment is negative (metallic tip has a lager work function than sample), the electric potential between sample and tip $\Phi_S = V_S - V_{CPD}$ will be much larger at the positive bias side (e.g., $\Phi_S \sim 1.7$ V in Fig. S6c, $\Phi_S \sim 1.4$ V in Extended Data Fig. 7f) than that at the negative bias side (e.g., $\Phi_S \sim 0.2$ V in Fig. S6c, $\Phi_S \sim 0.3$ V in Extended Data Fig. 7f). When the tip height increases, the electric field beneath tip tends to decrease as the tip and sample move farther apart, but prefer to increase because the V_{CPD} also declines at the same time. Therefore, Considering the competition between above two factors, the electric field could either increase or decrease as the tip lifts. According to the theoretical analysis in the *Domain manipulation* section in Methods, we found that due to the large potential difference between the two sides, the electric field under the tip is mainly dominated by the LCPD at the negative bias side, whereas at the positive bias side, it is mainly influenced by the tip-sample distance (Extended Data Fig. 7g,h). Thus, the switching bias required to trigger the same domain switch at a larger tip height has to decrease at the negative bias side, while increase at the positive bias side. Correspondingly, the two switching bias voltages have the negative and positive slope at respective polarity in the height dependent switching voltages diagram (Fig. S6c and Extended Data Fig. 7f), or we can say the slope is tip-sample potential Φ_S dependent. If there exists an exact pinning potential and a suitable LCPD that can perfectly counterbalance each other (Φ_S doesn't change with tip lift), it is possible to obtain a zero-slope diagram. In fact, as every domain wall has a different pinning potential (V_{SW} changes) and each tip has a different LCPD with the sample (V_{CPD} changes), the measured behaviour of switching voltages varies from case to case, resulting in different height-dependent slopes in various manipulations (i.e., in Fig. S6c and Extended Data Fig. 7f).

References

1. Chang, K. *et al.* Microscopic Manipulation of Ferroelectric Domains in SnSe Monolayers at Room Temperature. *Nano Lett.* **20**, 6590–6597 (2020).
2. Dangić, Đ., Fahy, S. & Savić, I. Giant thermoelectric power factor in charged ferroelectric domain walls of GeTe with Van Hove singularities. *npj Comput. Mater.* **6**, 1–8 (2020).
3. Chang, K. *et al.* Discovery of robust in-plane ferroelectricity in atomic-thick SnTe. *Science.* **353**, 274–278 (2016).
4. Liu, K., Lu, J., Picozzi, S., Bellaiche, L. & Xiang, H. Intrinsic Origin of Enhancement of Ferroelectricity in SnTe Ultrathin Films. *Phys. Rev. Lett.* **121**, 027601 (2018).
5. Damodaran, A. R., Breckenfeld, E., Chen, Z., Lee, S. & Martin, L. W. Enhancement of ferroelectric Curie temperature in BaTiO3 films via strain-induced defect dipole alignment. *Adv. Mater.* **26**, 6341–6347 (2014).
